# Exosome-derived circUPF2 enhances resistance to targeted therapy by redeploying ferroptosis sensitivity in hepatocellular carcinoma

**DOI:** 10.1186/s12951-024-02582-6

**Published:** 2024-05-30

**Authors:** Feng-Lin Dong, Zong-Zhen Xu, Ying-Qiao Wang, Tao Li, Xin Wang, Jie Li

**Affiliations:** 1 Department of General Surgery, The First Affiliated Hospital of Shandong First Medical University, Shandong Provincial Qianfoshan Hospital, No. 16766, Jingshi Road, Jinan, 250014 China; 2 Shandong First Medical University & Shandong Academy of Medical Sciences, Jinan, 250117 China; 3 Department of Hematology, The Third Affiliated Hospital of Shandong First Medical University, Jinan, 250014 China

**Keywords:** Hepatocellular carcinoma, Exosomal circRNA, SLC7A11, Ferroptosis

## Abstract

**Background:**

Advanced hepatocellular carcinoma (HCC) can be treated with sorafenib, which is the primary choice for targeted therapy. Nevertheless, the effectiveness of sorafenib is greatly restricted due to resistance. Research has shown that exosomes and circular RNAs play a vital role in the cancer’s malignant advancement. However, the significance of exosomal circular RNAs in the development of resistance to sorafenib in HCC remains uncertain.

**Methods:**

Ultracentrifugation was utilized to isolate exosomes (Exo-SR) from the sorafenib-resistant HCC cells’ culture medium. Transcriptome sequencing and differential expression gene analysis were used to identify the targets of Exo-SR action in HCC cells. To identify the targets of Exo-SR action in HCC cells, transcriptome sequencing and analysis of differential expression genes were employed. To evaluate the impact of exosomal circUPF2 on resistance to sorafenib in HCC, experiments involving gain-of-function and loss-of-function were conducted. RNA pull-down assays and mass spectrometry analysis were performed to identify the RNA-binding proteins interacting with circUPF2. RNA immunoprecipitation (RIP), RNA pull-down, electrophoretic mobility shift assay (EMSA), immunofluorescence (IF) -fluorescence in situ hybridization (FISH), and rescue assays were used to validate the interactions among circUPF2, IGF2BP2 and SLC7A11. Finally, a tumor xenograft assay was used to examine the biological functions and underlying mechanisms of Exo-SR and circUPF2 in vivo.

**Results:**

A novel exosomal circRNA, circUPF2, was identified and revealed to be significantly enriched in Exo-SR. Exosomes with enriched circUPF2 enhanced sorafenib resistance by promoting SLC7A11 expression and suppressing ferroptosis in HCC cells. Mechanistically, circUPF2 acts as a framework to enhance the creation of the circUPF2-IGF2BP2-SLC7A11 ternary complex contributing to the stabilization of SLC7A11 mRNA. Consequently, exosomal circUPF2 promotes SLC7A11 expression and enhances the function of system Xc- in HCC cells, leading to decreased sensitivity to ferroptosis and resistance to sorafenib.

**Conclusions:**

The resistance to sorafenib in HCC is facilitated by the exosomal circUPF2, which promotes the formation of the circUPF2-IGF2BP2-SLC7A11 ternary complex and increases the stability of SLC7A11 mRNA. Focusing on exosomal circUPF2 could potentially be an innovative approach for HCC treatment.

**Graphical Abstract:**

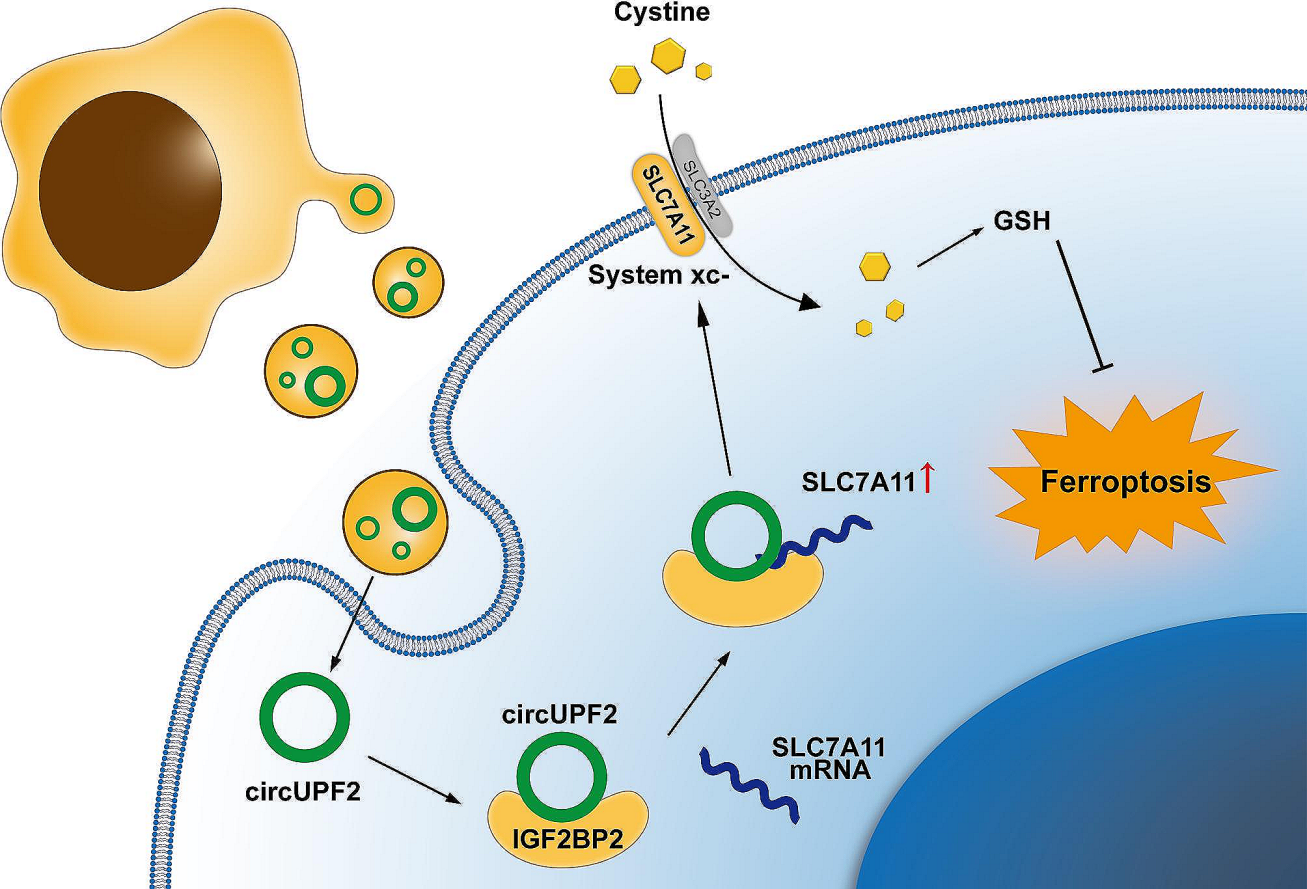

**Supplementary Information:**

The online version contains supplementary material available at 10.1186/s12951-024-02582-6.

## Introduction

Hepatocellular carcinoma (HCC) is the most common prevalent liver tumor, with an estimated incidence of over one million cases by 2025 [1, 2]. Less than 30% of patients with HCC are eligible for radical treatment upon initial diagnosis due to the gradual development of HCC tumors. Therefore, the administration of systemic antineoplastic treatment is crucial in managing patients at an advanced stage [3]. Sorafenib, the initial FDA-approved targeted treatment for advanced HCC, has been proven to provide a survival advantage in patients with advanced HCC from various nations and diverse liver disease backgrounds [4, 5]. Nevertheless, around 30% of individuals exhibit an inadequate response to sorafenib or acquire resistance following several months of treatment [6]. Multiple mechanisms, such as the tumor microenvironment, autophagy, epithelial-mesenchymal transition, and cancer stem cells, have been indicated as factors contributing to sorafenib resistance in HCC [7]. Moreover, given that sorafenib is among the limited number of tyrosine kinase inhibitors capable of triggering ferroptosis, there is a growing emphasis on understanding the resistance mechanism to sorafenib-induced ferroptosis in HCC [8, 9]. In HCC, epigenetic alterations often induce genomic instability prone to mutations. unlike genetic changes, epigenetic modifications are reversible and affect gene expression more extensively than genetic changes [10, 11]. Studies of epigenetic regulation and the involved molecular machinery are greatly contributing to the understanding of the mechanisms that underline HCC onset and heterogeneity, which may help to identify biomarkers for HCC diagnosis and prognosis, as well as future new targets for more efficacious therapeutic approaches. Thus, our study focuses on this aspect.

Ferroptosis, a type of programmed cell death, is controlled by various cellular metabolic events such as redox homeostasis, iron metabolism, mitochondrial function, and multiple disease-related signaling pathways [12, 13]. . It is distinguished by an excess of iron-dependent lipid reactive oxygen species (ROS) accumulation. The system Xc- cystine/glutamate antiporter, which consists of the subunits SLC7A11 and SLC3A2, is one of the regulators that imports cystine to produce the vital antioxidant peptide glutathione (GSH), which protects cells from death triggered by multiple oxidative stress condition [14]. The susceptibility of therapy-resistant cancer cells to ferroptosis is noteworthy, as it provides a promising alternative treatment for inducing the demise of cancer cells, particularly in malignancies that resist conventional therapies [15, 16]. As one of the important regulatory mechanisms of epigenetics, Non-coding RNAs (ncRNAs), including microRNAs (miRNAs), long non-coding RNAs (lncRNAs) and circular RNAs (circRNAs), are widely involved in the molecular control of ferroptosis in cancer via regulating gene expression at transcriptional and post-transcriptional levels [17]. Tumor cells can inhibit ferroptosis by removing their own strictly controlled ncRNA networks, which contributes to tumorigenesis and development. Consequently, our attention was directed towards investigating the process of developing resistance to therapy in HCC cells through targeting the cross talk between ncRNA and ferroptosis. This approach could potentially offer a novel tactic for enhancing the effectiveness of sorafenib treatment.

Exosomes, which have a size ranging from 30 to 150 nm, belong to a category of extracellular vesicles (EVs) that are discharged by viable cells [18, 19]. Studies conducted recently have shown that exosomes derived from tumors play a significant role in the development of treatment resistance in various types of cancers, including HCC [20, 21]. Exosomes play an important role in intercellular communication by transmitting nucleic acids, proteins and lipids from primitive cells to recipient cells [22]. Benefiting from its closed continuous loop structure, circRNA remains stable and is not easily degraded by RNase R, providing it with a competitive edge as a messenger molecule for intercellular communication [23, 24]. Generally, circRNAs function as endogenous sponges that adsorb microRNAs, and microRNAs silence target genes after transcription by attaching to the 3′-untranslated region (3′-UTR) of mRNAs [25]. Furthermore, research has shown that circRNAs can carry out their biological role through interaction with proteins [26]. However, the understanding of numerous circular RNAs and their respective roles in HCC remains incomplete, necessitating further investigations to elucidate this matter.

Here, we observed that exosomes derived from sorafenib-resistant Huh-7 (Huh-7-SR) cells could induce resistance to sorafenib in regular HCC cells. Further exploration of the functional molecules in exosomes led us to confirm that circUPF2 (circBase ID: hsa_circ_0017702) is critical for the development of sorafenib resistance in HCC. Mechanistic studies revealed that exosomal circUPF2 could act as a molecular scaffold to recruit IGF2BP2 and enhance the stability of downstream target SLC7A11 mRNA. Overexpression of SLC7A11 enhanced the function of system Xc- in HCC cells, resulting in a decreased sensitivity to ferroptosis and enhanced resistance to sorafenib. Although there are still some limitations in this study, such as insufficient exploration of the deeper mechanisms and few clinical samples, our findings still suggest that circUPF2 may serve as a promising therapeutic target to against HCC in the future.

## Materials and methods

### Cell lines

We purchased HCC cells (Huh7 and Hep3B) from the Typical Culture Reserve Center of China (Shanghai, China). Short tandem repeat DNA profiling was used to authenticate all cell lines. Huh-7 cells were cultured in DMEM (Dulbecco’s Modified Eagle’s Medium) from Gibco, Carlsbad, NY, USA, whereas Hep3B cells were cultured in MEM (Modified Eagle’s Medium) from Gibco. To establish the sorafenib-resistant HCC cell lines, Huh-7 cells were exposed to escalating amounts of sorafenib (from 0.5 to 10 µM) for a duration of six months. The selected cells (Huh-7-SR) were then cultivated in DMEM supplemented with a sorafenib concentration of 5µM to sustain the resistance.

### Co-culture systems and cell viability assay

Cell co-culture systems were established in a transwell chamber. Huh-7-SR cells were seeded in the upper compartment while Huh-7 or Hep3B cells were seeded in lower compartment. Cells in the system were grown in DMEM supplemented with 10% FBS that had been depleted of exosomes. After attachment, cells in the co-culture systems were treated with sorafenib (6µM) or DMSO. After 48 h, viable cells were quantified by Cell Counting Kit-8 (CCK-8, Dojindo Molecular Technologies, Kumamoto, Japan) assay following the manufacturer’s instructions. The optical density (OD) at 450 nm was measured using a Spectra Max 190 (Molecular Devices, Sunnyvale, CA, USA).

### Colony formation assay

HCC cells were counted and seeded at 150 cells per well in 24-well plates. After attachment, the cells would receive the appropriate treatment (sorafenib 6µM, erastin 5µM, or exosomes 10 µg prot/ml). After 14 days, cell colonies were washed with PBS, fixed with methanol for 15 min, and stained with crystal violet for 20 min. The colonies were imaged and counted.

### Samples of tissue and blood

Prior to collecting samples, the Medical Ethics Committee of The First Hospital Affiliated with Shandong First Medical University granted approval, and all subjects provided informed consent. The First Hospital Affiliated with Shandong First Medical University (located in Shandong Province, China) provided 31 blood samples, including 20 patients receiving sorafenib with no clinical progressive disease (group of non-PD) and 11 patients who had been treated with sorafenib but had an imaging-confirmed progressive disease (group of PD). In the meantime, a group of healthy individuals provided 20 blood samples, which served as negative controls. EDTA-containing tubes were used to collect all blood samples, and ultracentrifugation was employed to separate the circulating exosomes. To investigate the correlation between circUPF2 and SLC7A11 mRNA expressions, RNA extraction was performed on 36 HCC tissues collected from the First Hospital of Shandong First Medical University during the period of June 2022 to February 2023. Before RNA and protein extraction, all tissues were stored at − 80 °C.

### Isolation of exosomes

Huh-7-SR cells were grown in DMEM supplemented with 10% FBS that had been depleted of exosomes. Following collection, the medium underwent sequential centrifugation at 4 °C. The first centrifugation step was performed at 300 g for 10 min to eliminate the cell pellet. Subsequently, a second centrifugation step at 2000 g for 10 min was carried out to eliminate the dead cells. Finally, a third centrifugation step at 10,000 g for 10 min was conducted to eliminate the cell debris. Afterward, the supernatant was subjected to centrifugation at a force of 100,000 g for a duration of 90 min to acquire exosome precipitates, which were subsequently reconstituted in pre-chilled phosphate-buffered saline (PBS). The isolated exosomes were identified by nanoparticle tracking analysis (NTA, NanoSight NS300, Malvern, UK) and transmission electron microscopy (TEM, G2 spititi FEI, Tecnai, USA). A positive control was conducted using a standard sample of exosomes derived from HEK293 cells (Novus, USA), while a negative control was performed using Huh-7-SR cell lysate.

### Labeling and tracking of exosomes

The isolated exosomes were labeled using a PKH67 kit (MINI67-1KT, Sigma, USA) for green dye. After being labeled, the exosomes were co-incubated with HCC cells for a duration of 24 h. The cells were then examined and captured under a fluorescence microscope (Olympus FSX100, Tokyo, Japan).

### Extraction of RNA and conducting of PCR assay

TRIzol reagent (Invitrogen, Waltham, MA, USA) was used to extract total RNA from the tissues and cell lines, following the manufacturer’s instructions. To generate complementary DNA (cDNA), random primers and the FastKing RT Kits (TIANGEN Biotech, Beijing, China) for circRNA or mRNA were employed. The SYBR Green SuperReal PreMix Plus Kit (TIANGEN Biotech, Beijing, China) was used to perform a quantitative real-time PCR (qRT‒PCR) assay on a real-time fluorescence quantitative PCR system (CFX96, BioRad, Hercules, CA). GAPDH levels were used to normalize the disparities between circRNA and mRNA. Supplementary Table 1 contains the information about the primer details.

### RNA sequencing (RNA-seq) analysis and enrichment analysis

RNA integrity was assessed by quantifying the extracted RNA using NanoDrop ND-2000 (Thermo Scientific). The QIAGEN RNeasy Kit was used to purify the entire RNA, which was subsequently subjected to amplification and labeling with Cyanine-3-CTP (Cy3) dye. The sequencing library was 150PE which was determined by the Agilent 2100 Bioanalyzer using an Agilent DNA 1000 chip kit (Agilent Technologies, CA, USA). After elution, the Agilent Scanner G5761A (Agilent Technologies) was used to scan the initial images, and the Feature Extraction software (version 12.0.3.1, Agilent Technologies) was employed to extract the raw data. Quantile normalization and subsequent processing were performed using Genespring software (Agilent Technologies, version 14.8). The filtered normalized data underwent t-test to screen differentially expressed genes, based on the criteria of *P* value < 0.05 and log2 fold change (log2FC) > 1 in absolute value. Bioinformatics (www.bioinformatics.com.cn) is an online platform that provides free data analysis and visualization services. It was utilized for conducting enrichment analysis of Kyoto Encyclopedia of Genes and Genomes (KEGG) signal pathways, Gene Ontology (GO), and Gene Set Enrichment Analysis (GSEA).

### Cell transfection

OBiO Technology (Shanghai, China) designed and synthesized the overexpression vectors for hsa_circ_0017702 (OE-ciR). The hsa_circ_0017702 sequence was inserted into a pSLenti-EF1-F2A-Puro-CMV-S-circRNA-WPRE vector that contains front and rear circular frames. GenePharma (Shanghai, China) designed and produced small interfering RNAs (siRNAs) targeting hsa_circ_0017702 (si-ci17702) or other potential circRNAs, along with a negative control (NC). In order to achieve better transfection effectiveness, we utilized the jetPRIME agent (Polyplus Transfection, Illkirch, FRANCE) once the cells in the 6-well plates attained a confluence of 70%. The above vectors were validated for transfection efficiency using qRT‒PCR. Supplementary Table 2 contains the comprehensive lists of siRNA sequences.

### Western blotting

We performed Western blotting using antibodies against CD9 (Ab92726, 1/1000, Abcam), CD63 (Ab216130, 1/1000, Abcam), TSG101 (Ab125011, 1/1000, Abcam), Calnexin (Ab22595,1/1000, Abcam), CD81 (Ab79559, 1/500, Abcam), LAMP2 (Ab199946, 1/2000, Abcam), SLC7A11 (Ab175186, 1/1000, Abcam), GPX4 (67763-1-lg, 1/1000, Proteintech), IGF2BP2 (11601-1-AP, 1/1000, Proteintech, ), PCBP2 (Ab184962, 1/1000, Abcam), and GAPDH (10494-1-AP, 1/1000, Proteintech) as described in our previous publications [27].

### JC-1 staining

To measure the cells’ mitochondrial membrane potential (MMP), a JC-1 fluorescence probe kit (C2003S) from Beyotime in China was employed. JC-1 solution was introduced into the culture medium, followed by a 20-minute incubation period and subsequent washing with the JC-1 buffer solution. Images were captured utilizing a fluorescence microscope (Olympus FSX100, Tokyo, Japan).

### Assay for lipid reactive oxygen species (ROS)

We assessed the lipid ROS level in HCC cells by utilizing the C11-BODIPY dye (ABclonal, China, RM02821). Cells were exposed to 10 μm C11-BODIPY for a duration of 60 min, then collected, rinsed twice with PBS, and finally suspended in 500 µl of PBS.The dye’s fluorescence emission peak shifts from approximately 590 nm to around 510 nm due to the oxidation of its polyunsaturated butadienyl segment. The fluorescence emitted by C11-BODIPY was observed with a FACSCalibur flow cytometer (Becton Dickinson) and measured as the geometric mean (GeoMean).

### Detection of ferroptosis-related indicators

The levels of ferrous iron (Fe^2+^), glutathione (GSH), and cystine uptake in HCC cells were detected by the iron assay kit (APPLYGEN, China, E1042), GSH assay kit (Jiancheng Inc., China, A006-2-1), and Cystine Uptake Assay Kit (Dojindo, Japan, UP05) according to relevant manufacturers’ protocols.

### Evaluation of resistance to RNase R and the actinomycin D (ActD) assay

To identify circRNA, we conducted an RNase R resistance assay using RNase R (Geneseed, Guangzhou, China) as described in our previous reports [27]. Additionally, expressions of circUPF2, UPF2, and GAPDH compared to the control group was analyzed using the qRT‒PCR assay. The stability of RNA was assessed using the ActD assay from MedChem Express in New Jersey, USA. For the qRT‒PCR assay, the respective extraction of total RNA was carried out at 0, 2, 4, 6, or 8 h after administration.

### RNA immunoprecipitation (RIP) assay

The RIP assay was performed using the EZMagna RIP kit (Millipore, MA, USA) according to our previous reports [27]. A negative control was employed using Millipore’s Normal mouse IgG. Proteins that underwent immunoprecipitation were treated using proteinase K, and the corresponding RNA was isolated and examined using qRT‒PCR with divergent primers designed for circUPF2.

### RNA pull-down assay with MS2-labeled circUPF2

RNA pull-down assay using MS2-capturing protein (MS2-CP) was performed to identify the RNA-binding proteins (RBPs) associated with circUPF2. Firstly, two overexpression vectors were constructed by Geneseed Biotech (Guangzhou, China), including one carrying MS2-circUPF2 and the other carrying MS2-CP-Flag. Secondly, these two vectors were co-transfected into Huh-7 cells to induce MS2-circUPF2 and MS2-CP expression. After specific binding between MS2-labelled circUPF2 and MS2-CP, an anti-Flag antibody (A00170, GenScript, Nanjing, China) was used to pull down the MS2-CP-MS2-circUPF2 complex and qRT‒PCR or western blotting assays were performed to identify the captured products. Subsequently, the circUPF2 pull-down complex and its control were analyzed by mass spectrometry (Thermo Scientific Q Exactive, USA).

### Immunofluorescence (IF) assay and fluorescence in situ hybridization (FISH) assay

To examine the colocalization of circUPF2 and IGF2BP2 in HCC cells, IF-FISH assays were conducted. In short, Huh-7 and Hep3B cells were co-incubated with Exo-SR and then immobilized at ambient conditions using 4% paraformaldehyde. Next, the cells were covered with a circUPF2 probe labeled with FAM (Servicebio, Wuhan, China) and incubated at 37 °C for the entire night. Following three rounds of PBS washing, the cells were subjected to a 30-minute blocking step at 37 °C using 5% BSA and subsequently incubated overnight at 4 °C with an IGF2BP2 antibody (1:100, Abcam, ab313421). On the following day, the cells were rinsed using PBS and subsequently exposed to the appropriate secondary fluorescent antibody (Beyotime, Shanghai, China, A0516) for a duration of 30 min at 37 °C. This was then followed by sealing using parafilm that contained DAPI. RNA-FISH assays using FAM-labeled circUPF2 probe and Cy3-labeled SLC7A11 mRNA probe (Servicebio, Wuhan, China) were performed. According to our previous reports [27], we conducted RNA-FISH experiments using a circUPF2 probe labeled with FAM and an SLC7A11 mRNA probe labeled with Cy3 (Servicebio, Wuhan, China) to visualize the localization of circUPF2 and SLC7A11 mRNA in Huh-7 and Hep3B cells after co-culturing with Exo-SR. A confocal microscope (Nikon A1+, Minato, Japan) detected the signals from the probe.

### Electrophoretic mobility shift assay (EMSA)

Servicebio (Wuhan, China) provided biotin-labeled RNA oligonucleotides. The Chemiluminescent EMSA Kit (Beyotime, China, GS009) was utilized to perform the RNA EMSA assay, following the guidelines provided by the manufacturer. For EMSA, 4 µg of IGF2BP2 protein (Abcam, ab153107) was incubated with 1 pmol biotin-labeled probe in the binding buffer for 20 min at room temperature. For competitive EMSA, 100 pmol of unlabeled probes were added into the reaction mixture 20 min prior to the addition of a constant amount of the labeled positive probe. For super shift assays, recombinant proteins were preincubated with antibodies at a temperature of 0 °C for a duration of 20 min before labeled probes were added. The reaction mixtures were resolved on 6.5% non-denaturing polyacrylamide gels and transferred to nylon membranes (Beyotime, China). UV crosslinking was used to bind the RNA oligomers to the membrane, and the chemiluminescent imaging system was employed to detect the labeled probes.

### In vivo assay of tumor xenografts and exosomes

Prior to conducting the experiment, we acquired approval from the Institutional Animal Care and Use Committee of The First Hospital Affiliated with Shandong First Medical University. BALB/c nude mice were acquired from Gempharmatech Co., Ltd. (Certificate number: SCXK 2018-0008, Jiangsu, China). Every creature was housed in a sterile setting and provided with unlimited food. HCC cell line stably expressing luciferase (Huh-7-luc) was established using pSLenti-EF1-Luc2-F2A-Puro-CMV-MCS-WPRE (OBiO Technology, Shanghai, China, GL124). 20 mice were divided into 4 groups using a random number grouping method: G1 (DMSO + PBS), G2 (sorafenib + PBS), G3 (sorafenib + Exo-SR), and G4 (sorafenib + Exo-SR^si−ciR^). 5 × 10^6^ Huh-7-luc cells were injected subcutaneously into the right flank of mice. Tumor volume was measured and recorded every 2 days using the following formula: volume (mm^3^) = length (mm) × width^2^ (mm^2^)/2. When the tumor volume reached around 100 mm [3], the mice were given injections of DMSO/sorafenib every other day into the peritoneal cavity (i.p) at a dosage of 10 mg/kg. Additionally, they received intravenous injections (i.v) of PBS/Exo-SR (DiR labeled)/Exo-SR^si−ciR^ at a dosage of 200 µg. Tumors were visualized in vivo using the IVIS Imaging System (INVCLS1363, PerkinElmer, USA). Following a period of 20 days, the mice were euthanized, and the weight and the final volume of the tumors was measured.

### Immunohistochemistry (IHC) and TUNEL assay

A xenograft tumor biopsy was examined using antibodies to Ki-67 (ab15580, 1/100, Abcam) and SLC7A11 (DF12509, 1/100, Affinity) as described in our previous studies [27, 28]. The staining outcomes were measured utilizing Image-Pro Plus 6.0 (Media Cybernetics, USA) and presented as the proportion of cells exhibiting positivity (for Ki-67) or the average intensity (for SLC7A11). Cell apoptosis in xenograft tumor tissues was detected using the CF488 (Green) Tunel Cell Apoptosis Detection Kit (Servicebio, Wuhan, China) following the guidelines provided by the manufacturer.

### Statistical analysis

The data was counted and analyzed using GraphPad Prism 9 (GraphPad Software, LLC), and the mean ± standard deviation (SD) was used for data presentation. The differences between groups were analyzed using Student’s t-test, one-way analysis of variance (ANOVA), two-way ANOVA, and χ^2^ test. Linear regression analysis was used to determine the correlation between circUPF2 and SLC7A11 mRNA in human tissues.A significance level of less than 0.05 was deemed statistically significant.

## Results

### Exosomes obtained from Huh-7-SR cells enhanced resistance to sorafenib in HCC cells

**Fig. 1:**
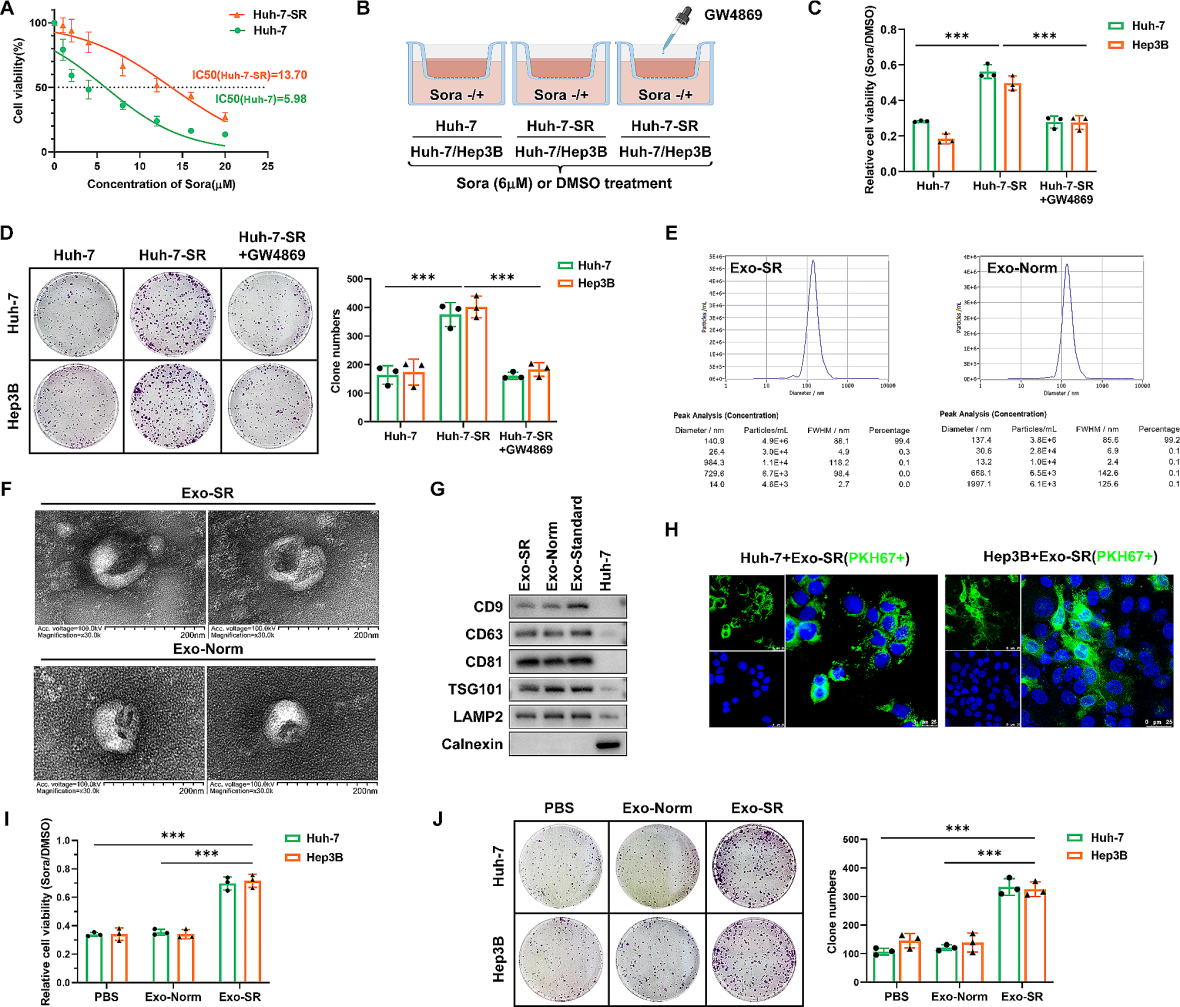
Exosomes derived from sorafenib-resistant Huh-7 cells induce an enhanced resistance to sorafenib in HCC cells. **(A)** Sorafenib-resistant cell line, Huh-7-SR, was established and confirmed by the IC50 value. **(B)** Co-culture systems were established to investigate the effects of sorafenib-resistant HCC cells on regular HCC cells. The group with additional GW4869 served as a negative control. **(C)** Cell viabilities of HCC cells co-cultured with Huh-7 or Huh-7-SR cells under sorafenib stress were evaluated via CCK-8 assays. **(D)** Assessment of cell proliferation using colony formation assay in Huh-7 and Hep3B cells with the indicated co-culture model. **(E)** Concentration and size of exosome were measured by NTA. Exosomes were isolated from the supernatant of the culture medium of Huh-7-SR (Exo-SR) and Huh-7 (Exo-Norm) cells by ultracentrifugation. **(F)** The iconic cup-shaped double-layer closed vesicle structure of isolated exosome was confirmed by TEM. **(G)** Western blotting analysis of exosomal markers, including CD9, CD63, CD81, LAMP2, TSG101 and Calnexin. An exosome standard from HEK293 cell line was analyzed as a positive control, while Huh-7-SR cell lysate was performed as a negative control. **(H)** The fluorescent signal of PKH67 labeled Exo-SR was captured in Huh-7 and Hep3B cells. **(I)** CCK-8 assays were performed to assess the cell viability of Huh-7 and Hep3B cells treated with sorafenib (6 µM) while co-cultured with the indicated exosomes. **(J)** Colony formation assays were performed to assess the cell proliferation of Huh-7 and Hep3B cells. The data are presented as the mean ± SD of at least three independent experiments. ***P < 0.001

To explore the role of exosomes in the development of resistance to sorafenib in HCC, we first cultured Huh-7 cells (IC50 = 5.98 µM) with different doses of sorafenib (0.5–10 µM) for 6 months, and established the sorafenib-resistant cell line Huh-7-SR (IC50 = 13.70 µM) (Fig. 1A). Next, a coculture system containing the sorafenib-resistant cell line and regular HCC cell lines was established to validate the interaction between different cell types under sorafenib stress, with the addition of the exosome-inhibited group using GW4869 [29] as a negative control (Fig. 1B). CCK-8 assays and colony formation assays were used to assess the viability and proliferation of HCC cells. These assays revealed that Huh-7 and Hep3B cells were able to obtain improved resistance to sorafenib by coculturing with Huh-7-SR cells, while GW4869 reversed this trend (Fig. 1C and D). This suggests that exosomes may play a critical role in the development of resistance to sorafenib in HCC cells. In order to investigate the process of exosome-mediated development of resistance to sorafenib in HCC, we isolated and characterized exosomes obtained from Huh-7-SR cells (Exo-SR) and Huh-7 cells (Exo-Norm). According to the NTA findings, the mean size of the isolated exosome samples was 140.9 nm, accounting for 99.4% (Exo-SR), and 137.4 nm, accounting for 99.2% (Exo-Norm) (Fig. 1E). In TEM images, the sample revealed cup-shaped nanoparticles that had a bilayer membrane structure, which is a distinctive characteristic of exosomes [30] (Fig. 1F). As reported as markers of exosomes [31], western blotting analysis confirmed the existence of CD9, CD63, CD81, TSG101 and LAMP2 and the absence of Calnexin in the extracted exosome samples. (Fig. 1G). The aforementioned findings indicate the successful isolation of exosomes. After that, exosomes were labeled with PKH67 and then added into the culture medium of Hep3B and Huh-7 cells for a duration of 24 h. Exosomes were taken up by HCC cells, as indicated by the detection of PKH67 fluorescent signal in both Hep3B and Huh-7 cells (Fig. 1H). Next, Huh-7 and Hep3B cells were cocultured with isolated Exo-SR or Exo-Norm in medium containing 6 µM sorafenib. The results of CCK-8 assays and colony formation assays confirmed that Exo-SR was responsible for inducing resistance to sorafenib in HCC cells (Fig. 1I and J).

### Exo-SR induces sorafenib resistance by suppressing ferroptosis in HCC cells

**Fig. 2:**
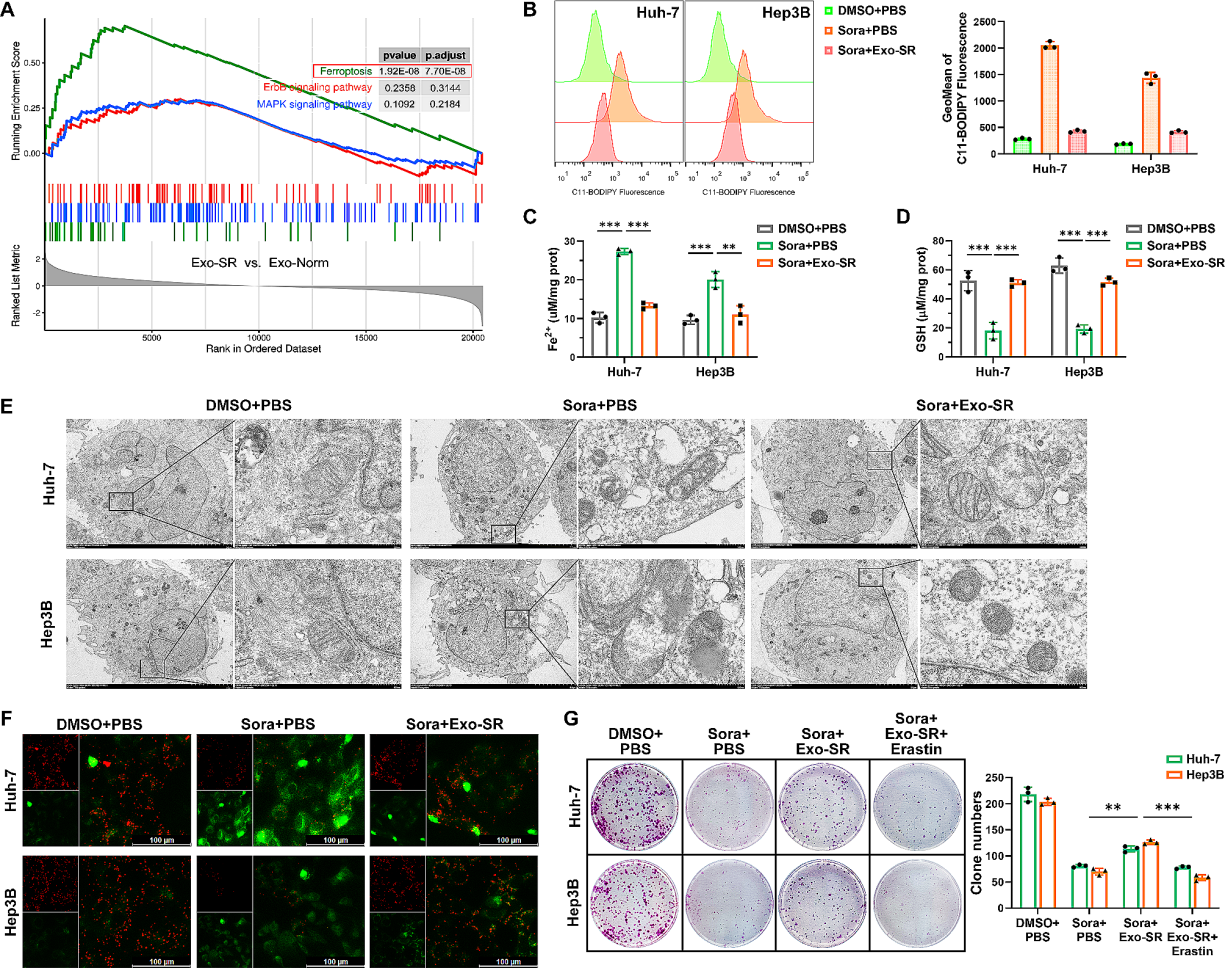
Exo-SR desensitized HCC cells to sorafenib-mediated ferroptosis. **(A)** The results of Gene Set Enrichment analysis (GSEA) showed that ferroptosis (the green line) was significantly enriched in Huh-7 cells co-cultured with Exo-SR. **(B)** Assessment of lipid ROS level in HCC cells co-cultured with Exo-SR by flow cytometry using C11-BODIPY. HCC cells were exposed to sorafenib (6 µM) or DMSO for 24 h. Results are quantified as geometric mean (GeoMean) fluorescence intensity. **(C), (D)** Quantification of Fe^2+^ and GSH levels in HCC cells treated with indicated conditions. (**E)** Visualization of ferroptotic morphological changes in HCC cells via TEM. **(F)** Assessment of mitochondrial membrane potential (MMP) using JC-1 staining. **(G)** The proliferation of HCC cells treated with indicated conditions was analyzed by colony formation assays. The data are presented as the mean ± SD of at least three independent experiments. **P < 0.01, ***P < 0.001

RNA-seq analysis was performed in Huh-7 cells cocultured with Exo-SR and Exo-Norm to investigate potential pathways involved in exosome-induced sorafenib resistance. Based on the integration analysis, 1749 differentially expressed genes (DEGs) (adjusted *P* < 0.05 and |log2FC| > 1.0) were shown by volcano plot (Supplementary Fig. 1A). Next, DEGs screened in the two groups of cells were subjected to KEGG pathway enrichment analysis and GO enrichment analysis. Notably, ferroptosis and molecules linked to ferroptosis were found to be enriched alongside their corresponding pathways, including cellular response to oxygen radical, autolysosome, and protein processing in endoplasmic reticulum (Supplementary Fig. 1B and 1 C). Sorafenib is known for suppressing tumor angiogenesis and inhibiting tumor cell proliferation. Therefore, we first validated Exo-SR whether Exo-SR was participates in angiogenesis. As shown in Supplementary Fig. 2A and 2B, Exo-SR showed no promotion on the viability of endothelial cells (HUVEC) in response to sorafenib stress and did not influence the expression of VEGF and HIF-1α in HCC cells. We thus excluded the ability of Exo-SR to induce resistance to sorafenib via suppressing angiogenesis in HCC. Furthermore, 3 KEGG pathways (ferroptosis, ErbB and MAPK signaling pathways) considered to be the main target of sorafenib in inhibiting HCC progression were analysis by GSEA [8, 32]. The results indicated that ferroptosis may be essential in the mechanism of exosome-induced sorafenib resistance in HCC (Fig. 2A). It is known that lipid peroxidation accumulation is a key event in ferroptosis, which is strongly associated with intracellular ferrous iron (Fe^2+^) accumulation and negatively modified by intracellular GSH [33]. Our results showed that Exo-SR was able to reverse the elevated levels of lipid ROS (Fig. 2B) and Fe^2+^ (Fig. 2C) in HCC cells caused by sorafenib and upregulate the intracellular GSH content (Fig. 2D), suggesting that Exo-SR functions in inducing sorafenib resistance by suppressing ferroptosis in HCC cells. Consistently, using TEM, we observed altered mitochondrial morphology (swelling, disorientation of cristae, and breakage) in sorafenib-treated HCC cells. In the Exo-SR coculture group, however, there were improvements in the ultrastructural morphology of mitochondria, as reflected by normalized crystal density and architecture (Fig. 2E). JC-1 staining indicated that Exo-SR could inhibit sorafenib induced JC-1 monomers and contribute to maintaining mitochondrial membrane potential (MMP) (Fig. 2F). Furthermore, the findings from colony formation experiments demonstrated that Exo-SR effectively reversed the suppressive impact of sorafenib on the proliferation of HCC cells, while this effect was neutralized when we additionally used erastin to reinduce ferroptosis in cells (Fig. 2G).

### Exo-SR protects HCC cells from ferroptosis by promoting SLC7A11 expression and maintaining the function of system Xc-

**Fig. 3:**
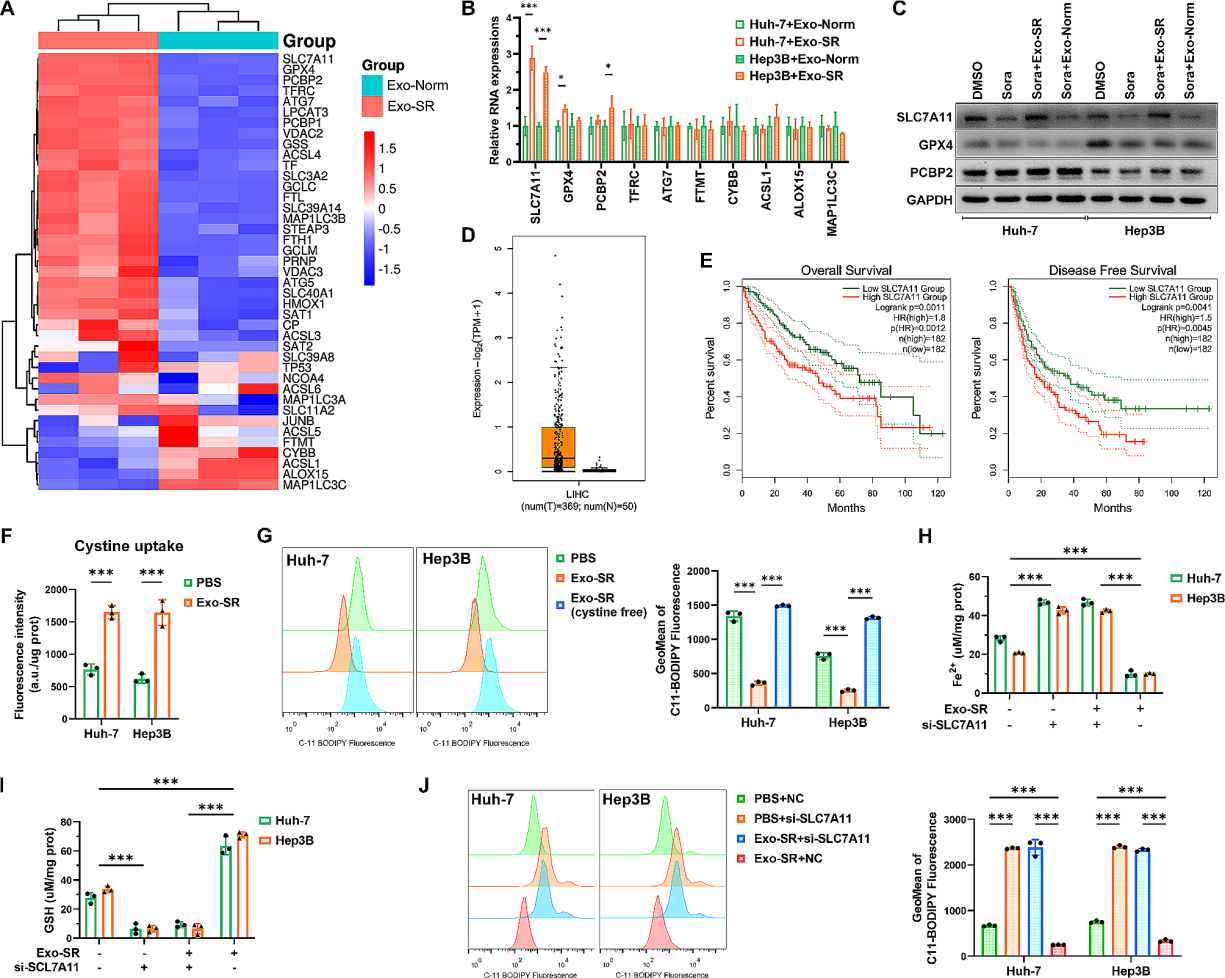
Exo-SR suppresses ferroptosis by promoting the expression of SLC7A11 in HCC cells. **(A)** Hierarchical cluster analysis showed differentially expressed genes (DEGs) related to ferroptosis in Huh-7 cells co-cultured with Exo-SR (*n* = 3) compared to those co-cultured with Exo-Norm (*n* = 3). The columns represent different cell samples. Each row indicates a gene, and the colors represent the abundance of the transcripts. **(B)** qRT-PCR analysis was applied to confirm the expression of the top 5 upregulated and 5 downregulated genes in HCC cells co-cultured with Exo-SR. **(C)** Western blotting assays were performed to evaluate the protein expression of SLC7A11, GPX4 and PCBP2 in the indicated groups, suggesting that Exo-SR significantly upregulated SLC7A11 expression in HCC cells. **(D)** The expression of SLC7A11 was dramatically more abundant in liver hepatocellular carcinoma (LIHC) than paired normal tissues in TCGA database. **(E)** The prognosis of abnormal SLC7A11 expression in LIHC patients from TCGA data. Results of Kaplan − Meier survival analysis showed a poor Overall survival (OS) and disease-free survival (DFS) in the high SLC7A11 group. **(F)** Activity of system Xc- in HCC cells was evaluated by cystine uptake assays. **(G)** Assessment of lipid ROS level in sorafenib-treated HCC cells, while the effect of Exo-SR on reducing lipid ROS level was blocked in cystine free culture medium. **(H), (I), (J)** Ferroptosis in sorafenib-stressed HCC cells with the indicated treatment was assessed by intracellular Fe^2+^, GSH and lipid ROS levels, and si-SLC7A11 transfection reversed Exo-SR-mediated suppression on ferroptosis. The data are presented as the mean ± SD of at least three independent experiments. *P < 0.05, ***P < 0.001

A heatmap was used to demonstrate differentially expressed genes related to ferroptosis in Exo-SR cocultured HCC cells screened via RNA-seq analysis (Fig. 3A). Upon further analysis using qRT‒PCR and western blotting assays, it was discovered that the presence of Exo-SR in HCC cells led to a notable elevation in the expression of SLC7A11 at both the mRNA and protein levels (Fig. 3B and C). Analysis of RNA sequencing data from The Cancer Genome Atlas (TCGA) project [34] revealed a rise in SLC7A11 levels in HCC tumors when compared to normal liver tissues (Fig. 3D). Moreover, the analysis of survival indicated that individuals with elevated levels of SLC7A11 experienced poorer overall survival and disease-free survival compared to those with lower levels of SLC7A11 (Fig. 3E). Considering that SLC7A11 acts as a major functional motif of system Xc- to regulate GSH synthesis via cystine uptake, we determined the effect of Exo-SR on the capacity of cystine uptake in HCC cells. As depicted in Fig. 3F, cells cocultured with Exo-SR exhibited increased cystine uptake ability compared to the control group. Correspondingly, Exo-SR also led to a decrease in lipid ROS levels in HCC cells, but this effect was eliminated when we replaced the culture medium with a cystine-free medium (Fig. 3G). Next, we suppressed SLC7A11 expression in HCC cells using si-SLC7A11 and cultured the cells in a medium containing 6 µM sorafenib. Fe^2+^, GSH and lipid ROS levels were quantified in HCC cells to assess the level of ferroptosis. The results suggested that artificially suppressing the expression of SLC7A11 in HCC cells would deprive the effect of Exo-SR in suppressing ferroptosis (Fig. 3H and J). All these findings indicate that Exo-SR protects HCC cells from ferroptosis by promoting SLC7A11 expression and maintaining the function of system Xc-.

### Exo-SR cells exhibit significantly enriched transcript levels of hsa_circ_0017702, which leads to sorafenib resistance in HCC cells

**Fig. 4:**
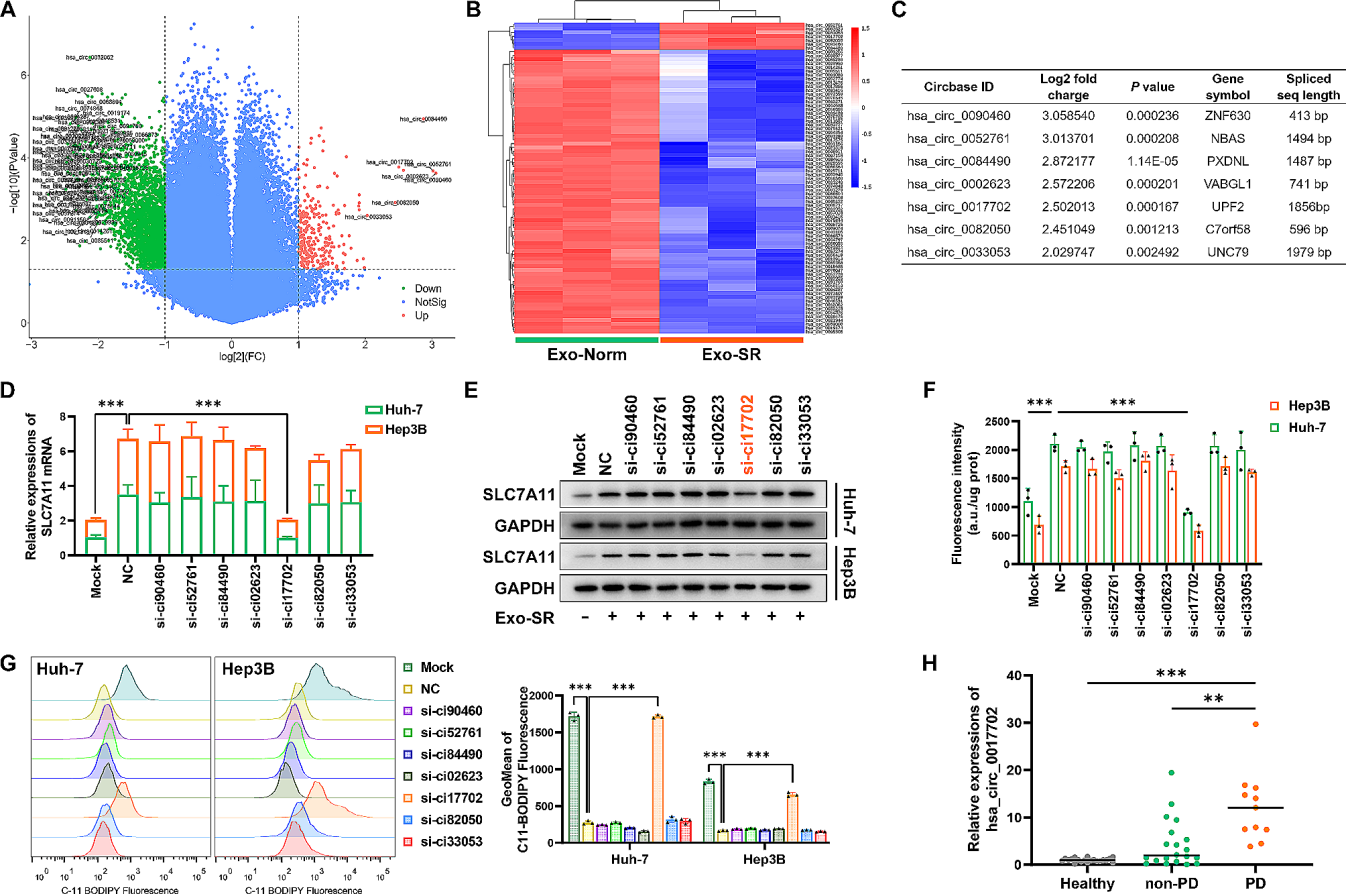
Exosomal hsa_circ_0017702 (circUPF2) regulated SLC7A11 gene expression in recipient cells. RNA-seq was performed in three pairs of Exo-SR and Exo-Norm to screen differentially expressed circRNAs. **(A)** Volcano plot illustrated differentially expressed circRNAs in three pairs of Exo-SR and Exo-Norm (fold change ≥ 2 and *P* < 0.05). Upregulated circRNAs were shown in red, while downregulated circRNAs were shown in green. **(B)** Heatmap showed clusters of circRNAs differentially expressed in three pairs of Exo-SR and Exo-Norm (fold change ≥ 4 and *P* < 0.05). **(C)** Table presented the information of the top 7 upregulated circRNAs in Exo-SR compared to Exo-Norm. **(D), (E)** qRT-PCR analysis and western blotting analysis were applied to assess the mRNA and protein expression of SLC7A11 in HCC cells co-cultured with exosomes derived from Huh-7-SR cells transfected with corresponding siRNA. **(F)** Assessment of the activity of system Xc- in HCC cells co-cultured with the indicated exosomes by cystine uptake assays. **(G)** Detection of lipid ROS level in sorafenib-treated HCC cells indicated that deprivation of exosomal function occurred when hsa_circ_0017702 in Exo-SR was knocked down. **(H)** qRT-PCR assays were performed to check the content of hsa_circ_0017702 in circulating exosomes isolated from the blood samples. Blood samples were obtained separately from healthy adult volunteers (Healthy), HCC patients receiving sorafenib with no clinical progressive disease (non-PD) and HCC patients who had been treated with sorafenib but had an imaging-confirmed progressive disease (PD). The data are presented as the mean ± SD of at least three independent experiments. **P < 0.01, ***P < 0.001

Mounting evidence suggests that circRNAs play a role in various cancer-related processes. Here, RNA-seq was conducted to acquire the expression profiles of circRNAs in exosomes. By performing RNA-seq on 3 corresponding samples of Exo-SR and Exo-Norm, a total of 88,689 circular RNAs were identified. Volcano plots were used to present the circRNAs that showed significant differential expression between the two groups. The cutoff criteria for inclusion were |log_2_FC| > 1.0 and *P* < 0.05. These plots included a total of 259 circRNAs that were significantly upregulated and 1610 circRNAs that were significantly downregulated (Fig. 4A). The heatmap displays the outcomes of hierarchical clustering using a cutoff criterion of |log_2_FC| > 2.0 and *P* < 0.05 (Fig. 4B). Seven circRNAs were identified as exhibiting the most significantly upregulated expression in Exo-SR, making them potential candidates for further investigation (Fig. 4C). In order to confirm the involvement of candidate circRNAs in the suppression of ferroptosis induced by exosomes, we developed siRNAs specifically targeting the splicing sequences of these circRNAs and introduced them into Huh-7-SR cells. After 48 h of transfection, exosomes (Exo-SR^si−ciR^) were isolated and then co-cultured with HCC cells. Using qRT‒PCR and western blotting assays we confirmed that the suppression of hsa_circ_0017702 in Exo-SR completely abolished its capacity to enhance SLC7A11 expression in HCC cells (Fig. 4D and E). Additionally, the elimination of hsa_circ_0017702 in Exo-SR abolished its role in sustaining the function of system Xc- (Fig. 4F) and reducing the levels of lipid ROS in HCC cells (Fig. 4G). Furthermore, qRT‒PCR tests were conducted to evaluate the concentration of hsa_circ_0017702 in exosomes obtained from the blood samples. The findings indicated that exosomes circulating in the PD group had notably elevated amounts of hsa_circ_0017702 compared to the healthy and non-PD groups. This suggests that exosomes may transmit hsa_circ_0017702 in HCC patients, particularly in those who experience disease progression following sorafenib treatment (Fig. 4H). Based on these results, hsa_circ_0017702 may play a crucial role in the development of sorafenib resistance in HCC cells induced by exosomes.

### hsa_circ_0017702 (circUPF2) in Exo-SR triggers the overexpression of SLC7A11, leading to the suppression of ferroptosis

**Fig. 5:**
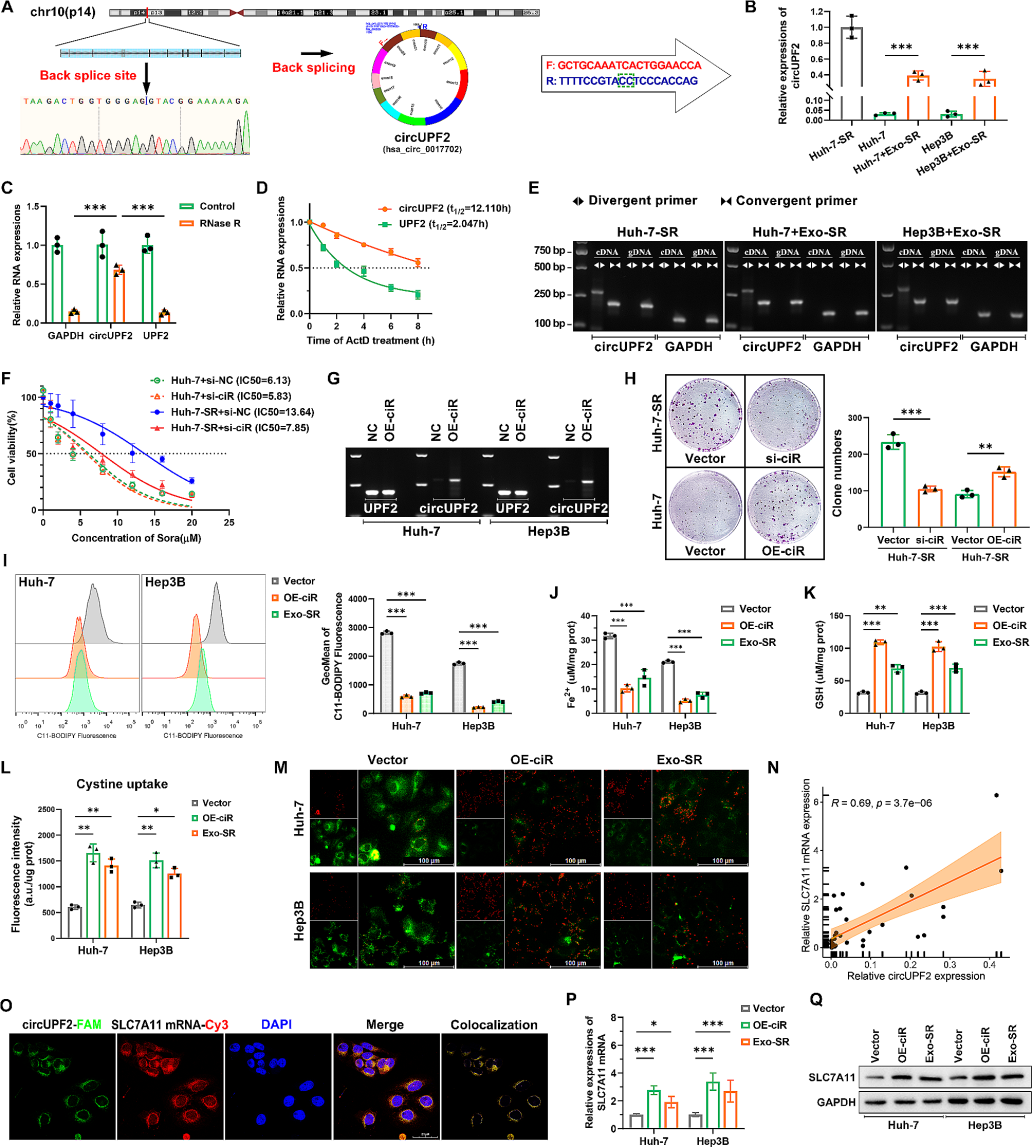
The characteristics of circUPF2 and the vital role of circUPF2 in suppressing sorafenib-mediated ferroptosis. **(A)** Schematic diagram of the circularized transcript generated from the UPF2 gene. The back-splicing site of circUPF2 was validated by Sanger sequencing, and the divergent primers were designed based on the spliced junction of circUPF2. **(B)** Relative expression changes of circUPF2 in the HCC cells after co-culture with Exo-SR were analyzed by qRT-PCR analysis. **(C)** RNase R was used to remove the linear transcripts from cellular extracts, leaving circRNAs behind. qRT‒PCR assays were applied to assess the resistance of RNAs to RNase R. **(D)** RNA decay assay evaluated the stability of circUPF2 and linear UPF2 by RT-qPCR after ActD (1 µg/ml) treatment. **(E)** CircUPF2 was amplified from both cDNA and gDNA samples by divergent or convergent primers; GAPDH was used as a negative control. Agarose gel electrophoresis visualized the products. **(F)** Huh-7 and Huh-7-SR cell lines transfected with si-circUPF2 (si-ciR) or negative control siRNA (si-NC) were treated with sorafenib and the values of IC50 were calculated via CCK-8 assays. **(G)** Overexpression vectors for circUPF2 (OE-ciR) were constructed. Agarose gel electrophoresis analysis was performed and confirmed that OE-ciR could accurately express circUPF2 but not UPF2 in HCC cells. **(H)** Cell proliferation was analyzed by colony formation assays in si-ciR transfected Huh-7-SR and OE-ciR transfected Huh-7 cells. Cells were treated with sorafenib (6 µM). **(I)** Detection of lipid ROS levels in OE-ciR or Vector transfected HCC cells and Exo-SR co-cultured HCC cells. **(J), (K)** Quantification of Fe^2+^ and GSH in the indicated group of HCC cells. **(L)** Activity of system Xc- in the indicated group of HCC cells was assessed by cystine uptake assays. **(M)** MMP of HCC cells in the indicated group was assessed by JC-1 staining. **(N)** Correlation analysis of circUPF2 and SLC7A11 expression analyzed by qRT-PCR assays in HCC tissues (*n* = 36). **(O)** RNA-FISH assay showed that circUPF2 is colocalized with SLC7A11 mRNA in the cytoplasm. **(P), (Q)** qRT-PCR and western blotting analysis were applied to assess the expression of SLC7A11 in the indicated group of HCC cells. The data are presented as the mean ± SD of at least three independent experiments. *P < 0.05, **P < 0.01, ***P < 0.001

According to circBase [35], hsa_circ_0017702 (circUPF2) is a circular RNA derived from exon 10 to exon 21 of the UPF2 gene on chr10, spanning approximately 1856 base pairs from position 11,971,863 to 12,009,453. Sanger sequencing additionally confirmed that the sequence near the junction site matched the findings from RNA-seq (Fig. 5A). In Huh-7-SR cells, results of qRT‒PCR assays indicated a notable upregulation in the relative expression of circUPF2, whereas Exo-SR notably enhanced the circUPF2 content in regular HCC cells (Fig. 5B). The analysis of RNase R resistance showed that circUPF2 exhibited a considerably greater resistance to RNase R compared to UPF2 and GAPDH (Fig. 5C). Upon the addition of ActD to Huh-7-SR cells during the specified time intervals, circUPF2 exhibited significantly greater stability compared to its linear counterpart (Fig. 5D). Furthermore, amplification was performed using either convergent or divergent primers targeting circUPF2, resulting in the detection of the circUPF2 band exclusively in the cDNA sample, but not in the genomic DNA, when divergent primers were utilized (Fig. 5E). The provided evidence indicated that circUPF2 is a stable circular transcript and can be effectively transported to regular HCC cells through Exo-SR. Subsequently, we employed siRNA (si-ciR) to inhibit the expression of circUPF2 and assessed the changes in sorafenib resistance of HCC cells using cell viability assays. The results depicted in Fig. 5F demonstrate that the reduction of circUPF2 effectively counteracted sorafenib resistance in Huh-7-SR cells. However, it did not have a notable impact on Huh-7 cells with lower circUPF2 expression, suggesting the crucial involvement of circUPF2 in sorafenib resistance specifically in Huh-7-SR cells. To mimic and explore the role of circUPF2 in HCC cells, we developed overexpression vectors of circUPF2 (OE-ciR) and confirmed that it can effectively increase the expression of circUPF2 in Huh-7 and Hep3B cells without interfering with the expression of its linear counterpart (Fig. 5G). Next, we overexpressed circUPF2 in Huh-7 cells using OE-ciR. We detected a significant increase in cellular resistance to sorafenib, whereas Huh-7-SR cells were sensitive to sorafenib when the expression of circUPF2 was suppressed (Fig. 5H).To further validate the impact of circUPF2 on ferroptosis in HCC cells, we further examined the changes in lipid ROS, Fe^2+^ and GSH levels in sorafenib-treated HCC cells, and the results showed that cellular ferroptosis was suppressed after overexpression of circUPF2, similar to the ingestion of Exo-SR(Fig. 5I and K). Consistently, HCC cells showed an elevated capacity for cystine uptake after overexpression of circUPF2, suggesting enhanced system Xc- function (Fig. 5L). Moreover, the mitochondrial activity in sorafenib-treated HCC cells was enhanced, as reflected in the restoration of the MMP (Fig. 5M). We also investigated the association between circUPF2 and SLC7A11 expression in HCC, considering our discovery that SLC7A11 is the primary regulatory focus of Exo-SR. The Spearman correlation analysis confirmed a positive correlation between circUPF2 and SLC7A11 mRNA expression in HCC tumor tissues (*P* < 0.001, R^2^ = 0.69) (Fig. 5N). Table 1 displays the correlation between the expression of circUPF2 in tissue samples and the clinical characteristics of cases. The FISH assay validated the colocalization of circUPF2 and SLC7A11 mRNA in the cytoplasm, indicating a potential interaction between the two (Fig. 5O). The findings from qRT‒PCR and western blotting assays revealed that overexpression of circUPF2 in HCC cells upregulated SLC7A11 expression, and this was also the case in cells cocultured with Exo-SR (Fig. 5P and Q). The aforementioned results suggest that circUPF2 plays a crucial role in Exo-SR by enhancing SLC7A11 expression and suppressing ferroptosis in HCC cells.

**Table 1:** Relationship between expression of circUPF2 in HCC tissues and clinical features of cases. (Appended at the end of the document text file)

	Clinical features				Relative RNA expression in tumor tissues		
Characteristics		Number		circUPF2 (mean ± SD)		*P* value	
**Gender**							
male		31		0.093 ± 0.126		0.053	
female		5		0.016 ± 0.017			
**Age**							
≤60		26		0.090 ± 0.131		0.547	
>60		10		0.063 ± 0.087			
**HBV**							
Negative		21		0.074 ± 0.125		0.600	
Positive		15		0.095 ± 0.116			
**AFP (ng/ml)**							
≤400		32		0.083 ± 0.120		0.913	
>400		4		0.076 ± 0.138			
**Child-Pugh stage**							
A		25		0.045 ± 0.079		**0.027** ^*^	
B		11		0.168 ± 0.154			
**Amount of tumor**							
Single		31		0.084 ± 0.126		0.835	
Multiple		5		0.072 ± 0.083			
**Tumor size**							
<3 cm		10		0.062 ± 0.132		0.526	
≥3 cm		26		0.091 ± 0.117			
**MVI**							
No		18		0.101 ± 0.131		0.353	
Yes		18		0.064 ± 0.109			
**ES nuclear grade**							
I - II		15		0.077 ± 0.098		0.801	
III - IV		21		0.087 ± 0.126			

*P < 0.05, **P < 0.01, ***P < 0.001;

AFP: alpha fetoprotein;

MVI: microvascular invasion;

ES nuclear grade: Edmondson-Steiner nuclear grade

### CircUPF2 interacts with IGF2BP2 as a scaffold complex

**Fig. 6:**
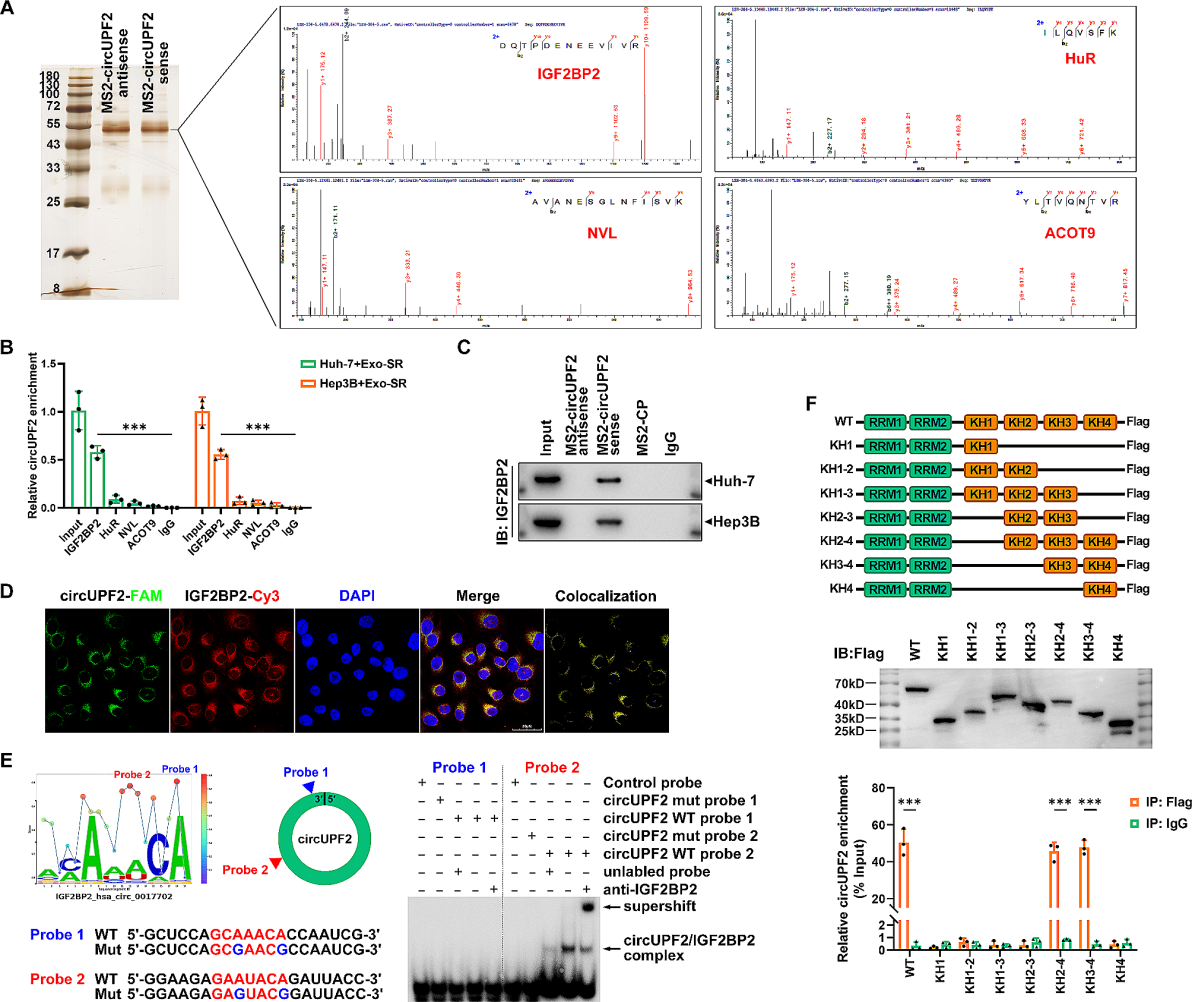
CircUPF2 interacts with IGF2BP2 as a scaffold complex. **(A)** Mass spectrometry (MS) analysis following RNA pull-down assays with MS2-labeled circUPF2 and an antisense probe as a negative control was performed to screen the potential circUPF2-interacting proteins in HCC cells. **(B)** Results of RIP assays verified IGF2BP2 as a putative circUPF2-binding protein. **(C)** Western blotting analysis following RNA pull-down assays further confirmed the interaction between circUPF2 and IGF2BP2 in Huh-7 and Hep 3B cells. **(D)** IF-FISH assay showed that circUPF2 is colocalized with IGF2BP2 in the cytoplasm. **(E)** Left, Schematic diagram showed the GCAAACA motif and the GAAUACA motif in circUPF2 and the RNA probe for RNA-EMSA assay. Right, RNA-EMSA assay showed the binding ability of purified IGF2BP2 with biotin-labeled oligonucleotides containing GAAUACA motif from circUPF2. **(F)** Top, schematic structures show RNA-binding domains within IGF2BP2 protein and a summary of IGF2BP2 truncations. Middle, immunoblot analysis with anti-FLAG of Huh-7 cells transfected with plasmids encoding FLAG-tagged WT or truncated IGF2BP2s. Bottom, relative enrichment representing circUPF2 levels associated with truncated IGF2BP2 relative to input control. The data are presented as the mean ± SD of at least three independent experiments. ***P < 0.001

In order to investigate the possible molecular mechanisms of circUPF2 in regulating ferroptosis and screen circUPF2-interacting proteins, we initially conducted RNA pull-down experiments, followed by mass spectrometry (MS) analysis using MS2-labeled circUPF2 and an antisense probe as a negative reference (Fig. 6A). Via RIP assays, we verified IGF2BP2 as a putative circUPF2-binding protein, which was reported to be essential for regulating mRNA stability [36] (Fig. 6B). Western blotting analysis following RNA pull-down assays further confirmed the interaction between circUPF2 and IGF2BP2 in Huh-7 and Hep 3B cells (Fig. 6C). The cytoplasmic colocalization of IGF2BP2 and exosomal circUPF2 was confirmed through IF-FISH assays, as shown in Fig. 6D. In the cytoplasm, circUPF2/IGF2BP2 forms an RNA-protein complex. Based on the RBPsuit database [37], we predicted that the GCAAACA motif (1269 nt-1275 nt) and the GAAUACA motif (1771 nt-1777 nt) in the circUPF2 sequence are putative binding motifs for IGF2BP2 (Fig. 6E, left). Furthermore, by performing EMSAs, we have verified that the GAAUACA motif within exon 17 of circUPF2 is crucial for the interaction with IGF2BP2. The motif specifically binds to IGF2BP2, as indicated by the findings of the Supershift experiments. The circUPF2-IGF2BP2 complex formation significantly decreased upon mutation of the GAAUACA motif (Fig. 6E, right). Afterwards, we explored the specific domain of IGF2BP2 that enables the interaction with circUPF2. The RIP experiments utilized IGF2BP2 mutants that had specific KH domains truncated, indicating that the di-domain KH3-4 is accountable for the binding of IGF2BP2 to circUPF2 (Fig. 6F). Collectively, these findings indicate that circUPF2 acts as a framework to trigger the creation of the circUPF2-IGF2BP2 complex through its GAAUACA pattern binding to the KH3-4 di-domain of IGF2BP2.

### The circUPF2-IGF2BP2 scaffold complex stabilizes SLC7A11 mRNA

**Fig. 7:**
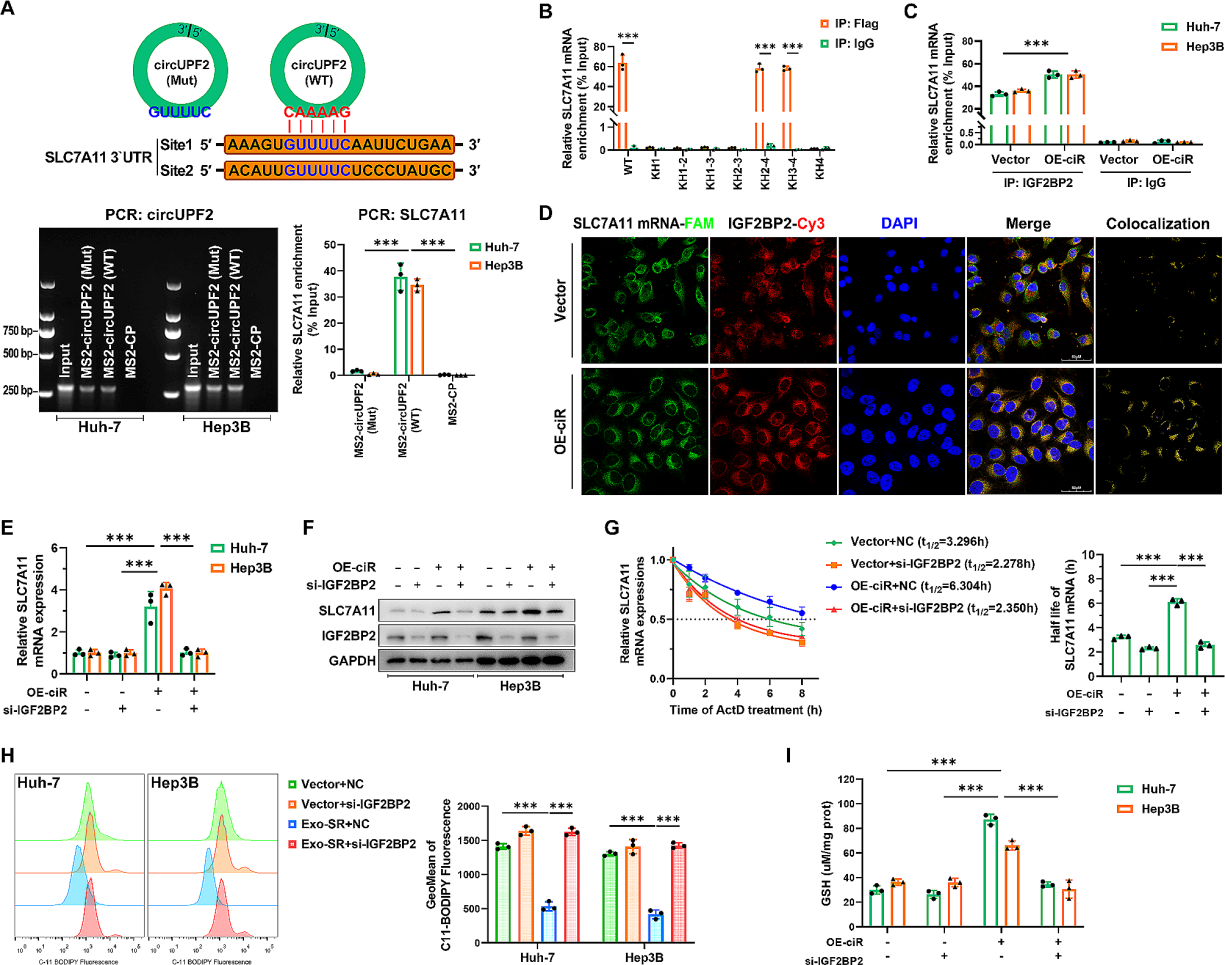
CircUPF2-IGF2BP2 scaffold complex stabilizes SLC7A11 mRNA. **(A)** Top, sequence BLAST analysis showed that circUPF2 directly targets the 3′UTR of SLC7A11 mRNA with high AU content. Bottom, MS2-CP pull-down efficiency of circUPF2 (Mut) and circUPF2 (WT) were detected by agarose gel electrophoresis (left panel), and the relative enrichment representing SLC7A11 mRNA levels associated with MS2-circUPF2 was quantized by qRT-PCR (right panel). **(B)** Relative enrichment indicated the enrichment of SLC7A11 associated with truncated IGF2BP2 protein complex compared to input control. IgG antibody served as a negative control. **(C)** RIP assays verified that overexpression of circUPF2 in HCC cells promotes the interaction between IGF2BP2 and SLC7A11 mRNA. **(D)** IF-FISH assays showed that the colocalization of IGF2BP2/SLC7A11 was increased upon overexpression of circUPF2. **(E), (F)** qRT-PCR analysis and Western blot analysis were used to assess the effects that overexpression of circUPF2 and knockdown of IGF2BP2 had on the expression of SLC7A11 at mRNA and protein levels in HCC cells. **(G)** Overexpression of circUPF2 significantly enhanced the stability of SLC7A11 mRNA in HCC cells, which was reversed by si-IGF2BP2. **(H), (I)** Levels of ferroptosis in HCC cells after overexpression of circUPF2 or knockdown of IGF2BP2 were assessed by detection of lipid ROS and quantification of GSH. The data are presented as the mean ± SD of at least three independent experiments. ***P < 0.001

Based on sequence BLAST analysis, we found that a CAAAAG motif inside circUPF2 can directly bind to the 3′-UTR of SLC7A11 (GUUUUC motif), which is rich in AU elements (Fig. 7A, top). Using RNA pull-down assays, we confirmed the interaction of circUPF2 and SLC7A11 mRNA (Fig. 7A, bottom). Afterwards, we examined the interaction between IGF2BP2 and SLC7A11 mRNA using RIP assays. The findings indicated that the KH3-4 di-domain of IGF2BP2 played a crucial role in its interaction with circUPF2 and SLC7A11 (Figs. 6F and 7B). Furthermore, it was discovered that the excessive overexpression of circUPF2 in HCC cells markedly increased the enrichment of SLC7A11 in IGF2BP2 immunoprecipitated fractions (Fig. 7C). The IF-FISH tests indicated that SLC7A11 mRNA and IGF2BP2 were colocalized in the cytoplasm and and the presence of the RNA‒protein complex was notably enhanced due to the overexpression of circUPF2 (Fig. 7D). In order to further examine the necessity of the circUPF2-IGF2BP2 scaffold complex formation for stabilizing SLC7A11 mRNA, we conducted simultaneous overexpression of circUPF2 and inhibition of IGF2BP2 expression in HCC cells. As depicted in Fig. 7E and F, the expression levels of SLC7A11 mRNA and protein were both upregulated by OE-ciR but reversed by si-IGF2BP2. The results of ActD assays suggested that the mRNA stability of SLC7A11 was significantly enhanced when circUPF2 was overexpressed, which required the presence of IGF2BP2 (Fig. 7G). Additionally, we observed that si-IGF2BP2 reversed the suppressive effect of OE-ciR on ferroptosis in HCC cells (Fig. 7H and I), which was consistent with the expression changes of SLC7A11. These findings demonstrate that circUPF2 promotes the interaction between IGF2BP2 and SLC7A11 mRNA, leading to increased stability of SLC7A11 mRNA by forming a ternary complex of circUPF2-IGF2BP2-SLC7A11 mRNA.

### Exo-SR significantly enhanced the resistance of HCC to sorafenib in vivo by delivering circUPF2

**Fig. 8:**
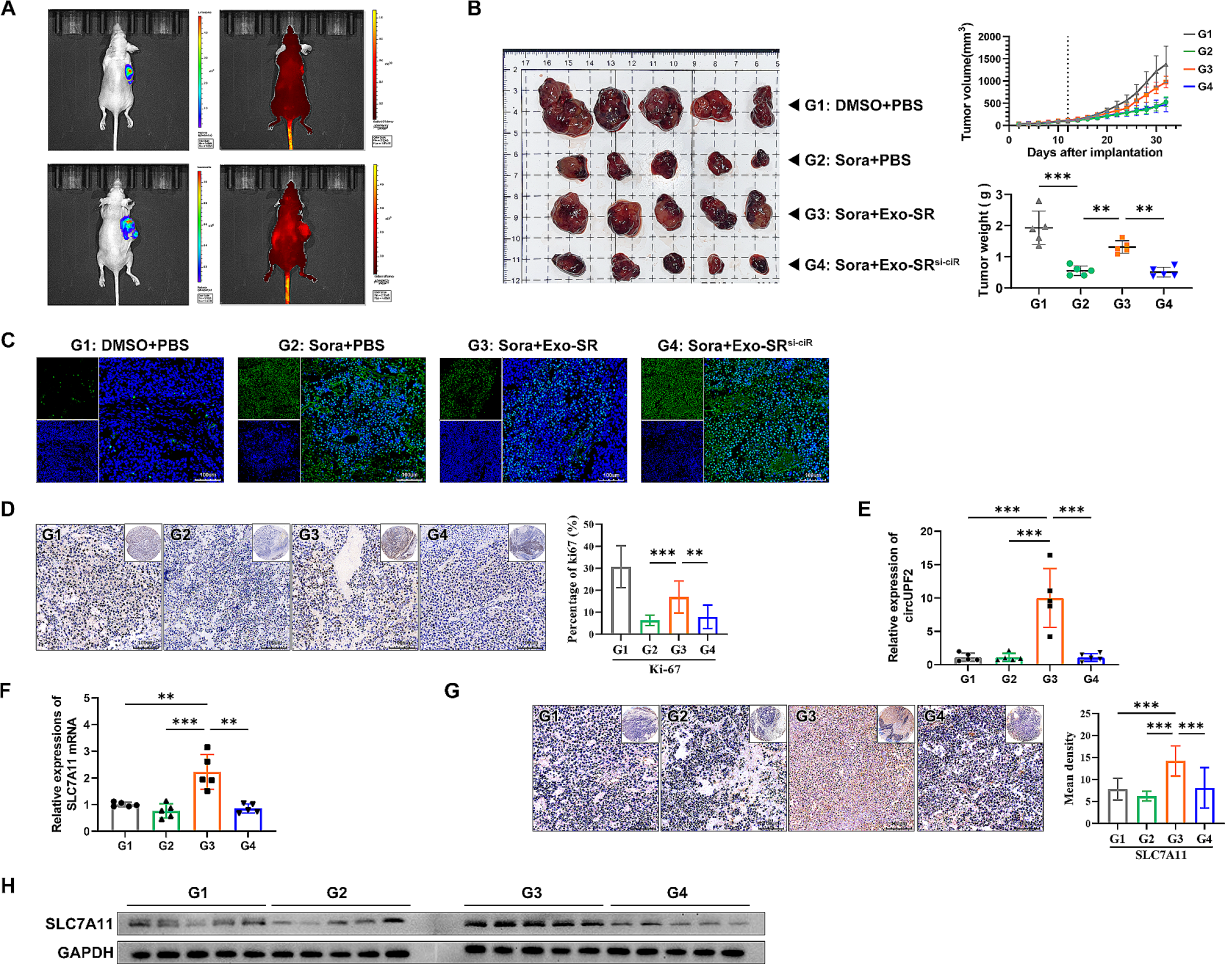
Exo-SR carrying circUPF2 suppresses sorafenib efficacy in vivo. **(A)** Luminescence images of representative xenograft tumors and distribution of DiR-labeled exosomes. **(B)** Xenograft tumors of nude mice 20 days after injection of Sorafenib/DMSO or Exo-SR/Exo-SR^si−ciR^ (*n* = 5 per group). Tumor growth curves were measured and plotted every two days after injection, while tumor weight was measured at the endpoint. **(C)** TUNEL staining was implemented to assess cell apoptosis in tumor tissues of the indicated groups. **(D)** Ki-67 expression in tumor tissues was detected by IHC staining to assess the proliferation of tumor cells. The results were quantified using Image-Pro Plus 6.0 and are shown as the percentage of positive cells. **(E), (F)** The expression of circUPF2 and SLC7A11 mRNA in xenograft tumor tissues was measured by qRT-PCR. **(G)** IHC staining was implemented to evaluate the expression of SLC7A11 in tumor tissues. The results were quantified using Image-Pro Plus 6.0 and are shown as the mean density. **(H)** Western blotting analysis of SLC7A11 expression showed elevated expression in xenograft tumor tissues of the G3 group(sorafenib + Exo-SR).

To explore the effects of exosomal circUPF2 in vivo, HCC xenograft models were established using Huh-7-luc cells. According to subgroups, mice received intraperitoneal injections of DMSO/sorafenib or intravenous injections of PBS/Exo-SR (DiR labeled)/Exo-SR^si−ciR^. In vivo fluorescence imaging showed a significant aggregation of DiR-labeled exosomes in the xenograft (Fig. 8A). The xenograft growth data confirmed the biological function of Exo-SR and circUPF2 in vivo. Compared with the sorafenib + PBS group, tumors in the sorafenib + Exo-SR group had a higher growth rate and greater weight, indicating a restricted efficacy of sorafenib. However, when we inhibited the expression of circUPF2 in Exo-SR (Exo-SR^si−ciR^), the tumors restored sensitivity to sorafenib, as evidenced by a lower tumor growth rate and lighter tumor weight (Fig. 8B). Cell apoptosis in tumor tissues was assessed using TUNEL staining. The findings showed that Exo-SR significantly inhibited cell apoptosis in sorafenib-treated HCC tumors, and this impact was reversed when circUPF2 expression in Exo-SR was suppressed (Fig. 8C). Identical results were also reflected in the proliferative capacity of the tumors characterized by the percentage of Ki-67 positive cells (Fig. 8D). Afterwards, we isolated total RNA from tumor tissues in order to examine the levels of circUPF2 and SLC7A11 using qRT‒PCR. The findings indicated that the levels of circUPF2 were notably elevated in tissues from the Exo-SR treated group (G3), and the expression of SLC7A11 was likewise significantly increased in the corresponding group (Fig. 8E and F). Furthermore, we verified that the levels of protein expression for SLC7A11 were markedly elevated following the administration of Exo-SR but not upon injection of exosomes with knockdown of circUPF2 (Fig. 8G and H). Nevertheless, the microvessel density (MVD) in tumor tissues was assessed by IHC assays using antibodies to CD31 and CD34 in order to clarify the effect of Exo-SR and circUPF2 on tumor angiogenesis in vivo. The results showed that the MVD in group G1 was significantly higher than that in the other three groups, but there was no significant difference in MVD between G2, G3 and G4 (Supplementary Fig. 2C). These results above suggest that neither Exo-SR nor circUPF2 could interfere with sorafenib’s suppression on tumor angiogenesis. Taken together, the above data suggest that Exo-SR promotes SLC7A11 expression and enhances the resistance of HCC to sorafenib in vivo by delivering circUPF2.

## Discussion

Currently, sorafenib is the primary medication for advanced HCC [4]. However, as its usage expands in clinical settings, acquired resistance to sorafenib has attracted widespread concern. This resistance significantly restricts the clinical benefit for HCC patients, and the specific mechanisms of this resistance remain unknown [38, 39]. It is crucial to conduct additional research on the mechanisms of sorafenib resistance in HCC in order to enhance the development of more efficient treatments. During this investigation, we established a sorafenib-resistant HCC cell line, Huh-7-SR, and these cells could stimulate sorafenib resistance in regular HCC cells when cocultured together. Notably, this process can be blocked by GW4869, which inhibits exosome release. This suggests that exosomes play an important role in the development of acquired resistance to sorafenib in HCC.

Growing evidence has demonstrated that exosomes can facilitate the growth, metastasis, and drug resistance of cancers by mediating crosstalk between tumor cells [40]. Our study found that the exosome-induced enhancement of resistance to sorafenib in HCC cells was accompanied by a reduction in intracellular lipid ROS and Fe^2+^ as well as an elevation in GSH levels, suggesting suppression of ferroptosis. Correspondingly, the results of GSEA analysis also revealed significant changes in the ferroptosis pathway in HCC cells after uptake of Exo-SR. Ferroptosis, distinct from apoptosis, necrosis, autophagy, and other types of cell death, is a form of controlled cellular demise. It is characterized by lipid peroxidation and involves intracellular iron accumulation [41]. Current research has identified that GPX4 is one of the core antioxidant defense enzymes for cellular resistance to ferroptosis, which utilizes GSH to detoxify lipid hydroperoxides [42, 43]. Cysteine is an important precursor for GSH synthesis and can limit GSH production, which is derived from the reduction of cystine uptaken from extracellular by system Xc- [43]. According to research, the clinical efficacy of sorafenib is partially dependent on its ability to induce ferroptosis by inhibiting the function of system Xc- in tumor cells, which is also closely related to the development of resistance to it [44]. System Xc- is composed of a transport module called xCT (SLC7A11) and a heavy chain component known as 4F2hc (SLC3A2). This system is responsible for the exchange of extracellular L-cystine with intracellular L-glutamate, which plays a crucial role in cellular regulation of redox levels via glutathione [12]. The overexpression of System Xc- has been verified in multiple tumors and is strongly linked to the advancement of tumors [45]. This was also confirmed in the present study by focusing on the function of system Xc- and analyzing the biochemical events related to ferroptosis in HCC cells upon sorafenib treatment. Importantly, our results indicated that Exo-SR was able to rescue the decrease in SLC7A11 expression induced by sorafenib, whereas interfering with SLC7A11 expression in HCC cells eliminated the suppressive effect of Exo-SR on ferroptosis. Taken together, these results reveal that Exo-SR protects HCC cells from ferroptosis through the promotion of SLC7A11 expression to maintain the function of system Xc-.

Exosomes are primarily dependent on their cargo molecules to perform the relevant functions [46]. Recently, exosomal circRNAs have attracted increasing attention. Here, we focused on the functional circRNAs delivered in exosomes and validated the substantial enrichment of several circRNAs in Exo-SR using RNA-seq analysis. With further loss-of-function studies, we identified a novel circRNA, circUPF2, enriched in Exo-SR that is responsible for exosome-induced sorafenib resistance in HCC cells. Moreover, our results also confirmed that SLC7A11 was the downstream target of circUPF2. Increased levels of circUPF2 in HCC cells elevate the expression of SLC7A11 and augment the activity of system Xc-, enabling HCC cells to exhibit reduced apoptosis and increased proliferation when faced with sorafenib-induced stress. The findings offer a fresh perspective on the transfer of exosomal contents during communication between HCC cells, prompting additional exploration into the intricate mechanisms by which circUPF2 controls ferroptosis.

Previous studies reported that circRNAs have the ability to engage with diverse proteins, resulting in the formation of a distinct circRNA-protein complex. This complex then affects the functioning of the associated proteins in different ways, as stated in previous studies [47]. Hence, we assessed the potential of circUPF2 to interact with particular proteins and regulate their activities. After conducting the RNA pull-down assay, mass spectrometry analysis, RIP, and EMSAs, it was verified that circUPF2 effectively interacts with IGF2BP2 in the cytoplasm. IGF2BP2 was previously reported to be an RNA-binding protein for mRNA stabilization [48], and in this study, RNA-FISH assays verified that the exosomal circUPF2 and SLC7A11 mRNA colocalized in the cytoplasm. Thus, our hypothesis was that circUPF2 acts as a framework to facilitate the attachment of IGF2BP2 to SLC7A11 mRNA, leading to an enhancement in mRNA durability, and we confirmed this assumption. First, we confirmed the physical interactions of both circUPF2 and IGF2BP2 with SLC7A11 mRNA by performing RNA pull-down and RIP assays and validated the relevant binding domains. Then, we demonstrated the ability of circUPF2 to significantly promote the interaction of IGF2BP2 and SLC7A11 mRNA in HCC cells using an overexpression vector. Likewise, the findings from IF-FISH assays revealed that circUPF2 significantly increased the colocalization of the SLC7A11-IGF2BP2 RNA-protein complex. Next, we performed ActD assays and verified that circUPF2 significantly extends the half-life of intracellular SLC7A11 mRNA in a manner dependent on IGF2BP2, which leads to the suppression of ferroptosis in HCC cells. Finally, we demonstrated that circUPF2 promoted the expression of SLC7A11 in vivo, and exosomes carrying circUPF2 dramatically attenuated the suppressive effect of sorafenib on HCC xenograft tumors.

Our study provides the mechanism by which exosomal circUPF2 induces sorafenib resistance in HCC and extends evidence for further studies of exosomal circRNAs. Nevertheless, other mechanisms are present in cells to combat ferroptosis besides system Xc–GSH-GPX4. In GPX4-deficient cells, ferroptosis suppressor protein 1 (FSP1) translocates from mitochondria to the cell membrane and catalyses the regeneration of coenzyme Q10 (CoQ10) using NAD(P)H, which can trap lipid peroxyl radicals that mediate lipid peroxidation [49, 50]. Besides, GTP cyclohydrolase-1 (GCH1) was demonstrated to inhibit cytosolic lipid peroxidation by catalyzing the synthesis of BH4 and the GCH1-BH4 pathway acts as an endogenous antioxidant pathway to inhibit ferroptosis through a mechanism independent of the GPX4/glutathione system [51, 52]. Generally, these mechanisms ultimately suppress ferroptosis via the inhibition of lipid peroxidation, as is the role of Exo-SR identified in our study. The difference, however, is that the circUPF2-mediated suppression of ferroptosis is dependent on adequate exogenous cystine and the proper functioning of GPX4. These mechanisms complement each other and provide ideas for a comprehensive view of the influence of circUPF2 on ferroptosis and for exploring target of intervention. However, there are still constraints in this research. Further exploration is needed to understand the detailed mechanism of how IGF2BP2 recognizes the binding structural domains on circUPF2 and SLC7A11 mRNA sequences. Furthermore, it is imperative to conduct additional research on the clinical relevance of exosomal circUPF2 in both tissue and serum samples from a larger population of HCC patients, preferably in a multicenter setting. Clinicians would greatly benefit from a deeper comprehension of the diagnostic and prognostic significance of circUPF2, as it would aid in the advancement of proactive therapeutic approaches.

## Conclusion

This paper discovered a new circRNA, circUPF2, in exosomes derived from sorafenib-resistant HCC cells. This study also presented the initial comprehensive proof that exosomal circUPF2 plays a crucial role as an oncogenic circular RNA in the progression of acquired sorafenib resistance in HCC. The formation of a circUPF2-IGF2BP2-SLC7A11 ternary complex is crucial for the stabilization of SLC7A11 mRNA, leading to the suppression of ferroptosis in HCC cells. The results of our study provide insight into the biological roles of exosomal circUPF2 and reveal its scaffolding mechanism, presenting a potential biomarker and target for treating sorafenib-resistant HCC.

### Electronic supplementary material

Below is the link to the electronic supplementary material.

Supplementary Material 1

Supplementary Material 2

## Data Availability

The datasets used and/or analyzed during the current study are available from the corresponding author upon reasonable request.

## Abbreviations

EMSA: Electrophoretic mobility shift assay
EV: extracellular vesicle
Exo-Norm: Exosomes derived from Huh-7 cells
Exo-SR: Exosomes derived from sorafenib-resistant Huh-7 cells
FISH: fluorescence in situ hybridization
GO: Gene Ontology
GSEA: Gene Set Enrichment Analysis
HCC: Hepatocellular carcinoma
Huh-7-SR: sorafenib-resistant Huh-7
IF: immunofluorescence
IHC: Immunohistochemistry
KEGG: Kyoto Encyclopedia of Genes and Genomes
MMP: mitochondrial membrane potential
MS2-CP: MS2-capturing protein
NC: negative control
NTA: Nanoparticle tracking analysis
OE-ciR: overexpression vectors for hsa_circ_0017702 (circUPF2)
si-ciR: small interfering RNAs against hsa_circ_0017702 (circUPF2)
RBP: RNA-binding protein
RIP: RNA immunoprecipitation
ROS: reactive oxygen species
TCGA: The Cancer Genome Atlas
TEM: transmission electron microscopy
