# Supplementary material for: Exosome-derived circUPF2 enhances resistance to targeted therapy by redeploying ferroptosis sensitivity in hepatocellular carcinoma
Source: J Nanobiotechnology. 2024 May 30;22:298. doi: 10.1186/s12951-024-02582-6 (PMC11137910; doi:10.1186/s12951-024-02582-6)
Supplement: Supplementary file 1 — Supplementary Material 1 [file 12951_2024_2582_MOESM1_ESM.docx]

**Supplementary Table 1:** **qRT-PCR primer sequences**

| **Primer name** | **Primer sequence** |
| --- | --- |
| GAPDH-F | GTCTTCACCACCATGGAGAA |
| GAPDH-R | AAGCAGTTGGTGGTGCAG |
| hsa_circ_0017702 (circUPF2) di-F | GCTGCAAATCACTGGAACCA |
| hsa_circ_0017702 (circUPF2) di-R | TTTTCCGTACCTCCCACCAG |
| hsa_circ_0017702 con-F | TCCACGTTGTGGATGGAGTG |
| hsa_circ_0017702 con-R | GTCCAGGGAACTTGGAGAGC |
| hsa_circ_0090460 di-F | TGAATCCTGCTCAGAAGACC |
| hsa_circ_0090460 di-R | GAACTTCGTCCTGGGTAGAT |
| hsa_circ_0052761 di-F | GCTAAAGATTGGTGCGAGGA |
| hsa_circ_0052761 di-R | TCATCATAGCCTGGCTGC |
| hsa_circ_0084490 di-F | GGAGAACCACAGCCCATAAT |
| hsa_circ_0084490 di-R | CAGGTACCCGTGACTGT |
| hsa_circ_0002623 di-F | CTTGTGGATGCCCTCCTCTT |
| hsa_circ_0002623 di-R | TCTAGTTCTTTCCCTCAGGTGT |
| hsa_circ_0082050 di-F | TAGAGAAGACCGCCCAAGTC |
| hsa_circ_0082050 di-R | AGAATTCCTTTCATGTCCCCA |
| hsa_circ_0033053 di-F | ACGCGTTCATCTTTGCTCAG |
| hsa_circ_0033053 di-R | AGTAGAAGGATCACTTTGTTATGGG |
| SLC7A11-F | ACGGTGGTGTGTTTGCTGTCTC |
| SLC7A11-R | GCTGGTAGAGGAGTGTGCTTGC |
| GPX4-F | CATCATCGGAAAGGAGGGCTTGAC |
| GPX4-R | GCATGGATGGTGACAGGCTTCTC |
| PCBP2-F | TCTGCGTGGTCATGTTGGAG |
| PCBP2-R | TGCATCCAAACCTGCCCAATA |
| TFRC-F | GGACGCGCTAGTGTTCTTCT |
| TFRC-R | CATCTACTTGCCGAGCCAGG |
| ATG7-F | TGTGCCTCACCAGGTTCTTG |
| ATG7-R | ATCATCGCTCATGTCCCAGA |
| UPF2-F | TCTGATAGACAAGGCAGCAA |
| UPF2-R | TTGATCTGGTCCTTTTTCCGT |

**Supplementary Table 2: Sequences of siRNA.**

| **Oligo name** | **Sequence** |
| --- | --- |
| si-circ_0017702 (si-ciR) | 5′- AGACTGGTGGGAGGTACGGAA -3′ |
| si-circ_0090460 | 5′- CCCAGGACGAAGTTCCGGAAA -3′ |
| si-circ_0052761 | 5′- CGGCAGCCAGGCTATGATGAC -3′ |
| si-circ_0084490 | 5′- CAGTCACGGGTACCTGTATAA -3′ |
| si-circ_0002623 | 5′- TGGGACACCTGAGGGAAAGAA -3′ |
| si-circ_0082050 | 5′- TCTGGGGACATGAAAGGAATT -3′ |
| si-circ_0033053 | 5′- AAGTGATCCTTCTACTAAAGG -3′ |
| si-NC | 5′- UUCUCCGAACGUGUCACGUTT -3′ |
| si-SLC7A11 | 5′- GGGUGGAACUCCUCAUAAUTT -3′ |
| si-IGF2BP2 | 5′- GCCGCAUGAUUCUUGAAAUTT -3′ |

**Supplementary Table 3: Sequences of** **probe for RNA-FISH.**

| **Oligo name** | **Sequence** |
| --- | --- |
| circUPF2 | 5′- TCCGTACCTCCCACCAGTC -3′ |
| SLC7A11 | 5′- GGATGAAACATACTCTGGCC -3′ |

**Supplementary Table 4:** **Sequences of circUPF2 probe for EMSA.**

| **Oligo name** | **Sequence (5'-3')** |
| --- | --- |
| circUPF2-WT probe 1 | Biotin - GCTCCAGCAAACACCAATCG -biotin |
| circUPF2-mut probe 1 | Biotin - GCTCCAGCGAACGCCAATCG -biotin |
| circUPF2-WT probe 2 | Biotin - GGAAGAGAATACAGATTACC -biotin |
| circUPF2-mut probe 2 | Biotin - GGAAGAGAGTACGGATTACC -biotin |
