# Supplementary material for: Exosome-derived circUPF2 enhances resistance to targeted therapy by redeploying ferroptosis sensitivity in hepatocellular carcinoma
Source: J Nanobiotechnology. 2024 May 30;22:298. doi: 10.1186/s12951-024-02582-6 (PMC11137910; doi:10.1186/s12951-024-02582-6)

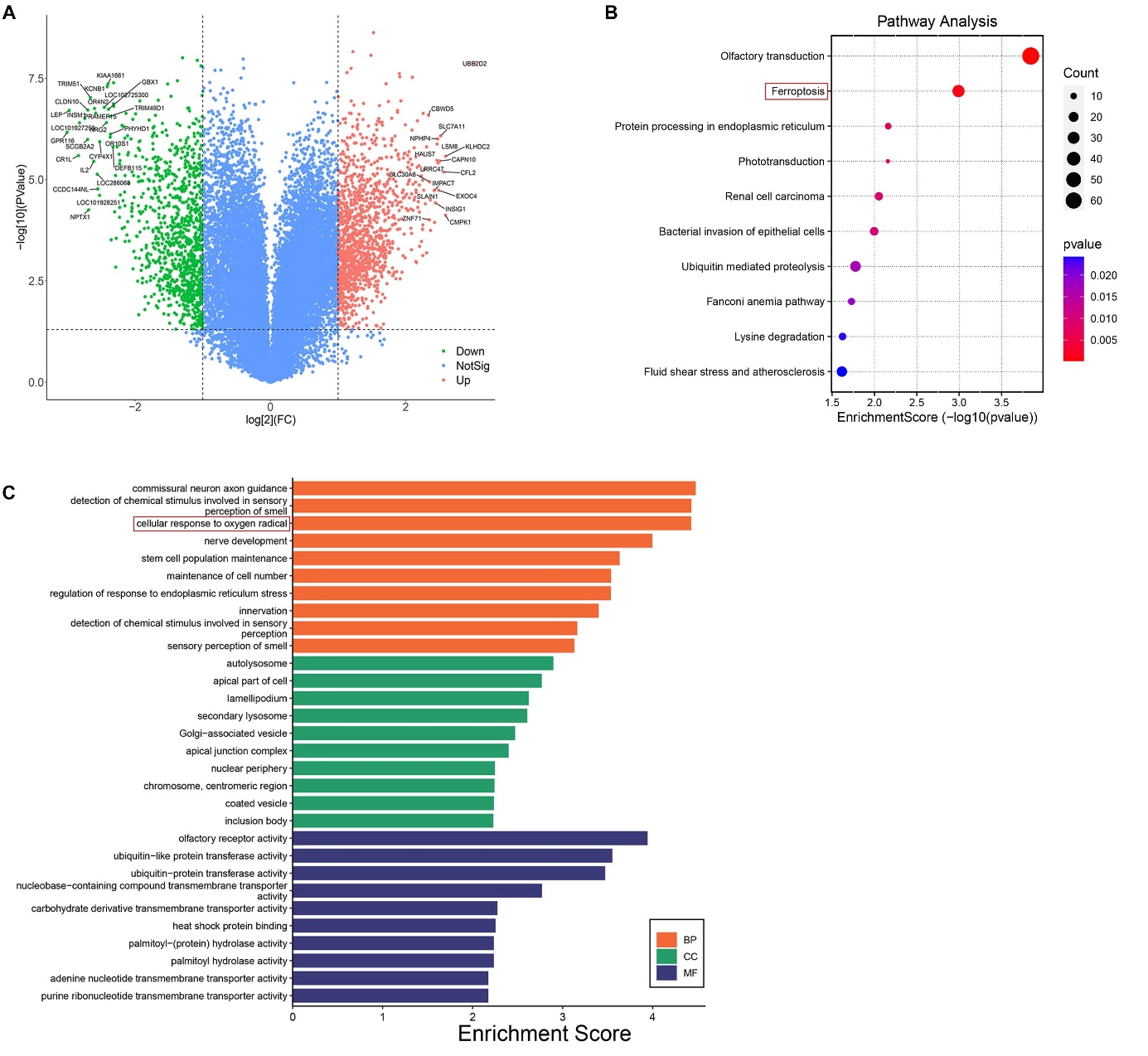


**Supplementary Figure1 (A)** Volcano plot illustrated differentially expressed genes (DEGs) in Huh-7 cells co-cultured with Exo-SR (n=3) compared to those co-cultured with Exo-Norm (n=3). Upregulated genes were shown in red, while downregulated genes were shown in green. **(B)**, **(C)** GO and KEGG analyses of significant DEGs. The results showed cellular response to oxygen radical and ferroptosis pathway were significantly enriched.


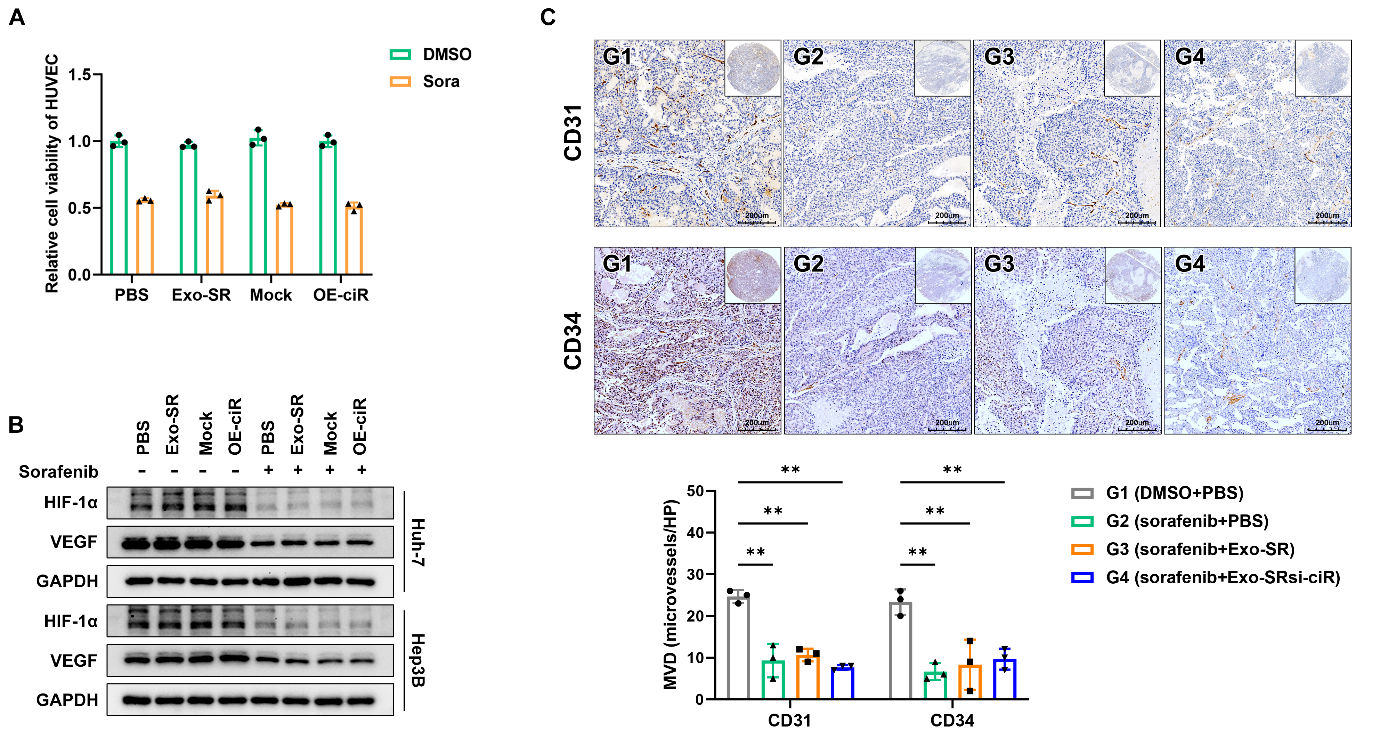
**Supplementary Figure 2 Verification of the effect of Exo-SR and circUPF2 on HCC tumor angiogenesis. (A)** The result of CCK-8 assay showed that neither Exo-SR nor circUPF2 could promote the proliferation of HUVEC in vitro. **(B)** The effects of Exo-SR or circUPF2 on the expression of VEGF and HIF-1α in HCC cells were also examined by western blotting assays, which indicated that neither Exo-SR nor circUPF2 had a significant effect on VEGF or HIF-1α expression. **(C)** The microvessel density (MVD) in xenograft tumor tissues was assessed by IHC assays using antibodies to CD31 and CD34. The results showed that the MVD of tumor tissues in group G1 (DMSO+PBS) was significantly higher than that in G2 (sorafenib+PBS), G3 (sorafenib+Exo-SR) and G4 (sorafenib+Exo-SRsi-ciR), but there was no significant difference in MVD between G2, G3 and G4.

**Supplementary Figure 3 The results of the RNA-seq data analysis related to lncRNAs in exosomes. (A)** Volcano plot illustrated differentially expressed lncRNAs in three pairs of Exo-SR and Exo-Norm (fold change ≥ 2 and *P* < 0.05). Upregulated lncRNAs were shown in red, while downregulated ones were shown in green. (B) The heatmap showed clusters of lncRNAs differentially expressed in three pairs of Exo-SR and Exo-Norm (fold change ≥ 4 and *P* < 0.05).
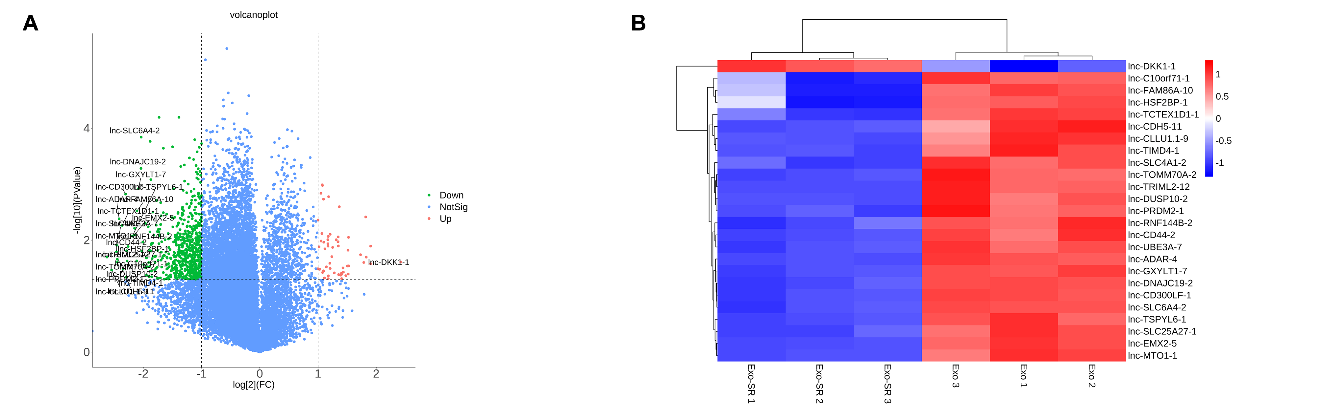

Supplement: Supplementary file 2 — Supplementary Material 2 [file 12951_2024_2582_MOESM2_ESM.docx]
